# Learning-based inference of longitudinal image changes: Applications in embryo development, wound healing, and aging brain

**DOI:** 10.1073/pnas.2411492122

**Published:** 2025-02-20

**Authors:** Heejong Kim, Batuhan K. Karaman, Qingyu Zhao, Alan Q. Wang, Mert R. Sabuncu, for the Alzheimer’s Disease Neuroimaging Initiative

**Affiliations:** ^a^Artificial Intelligence in Radiology, Radiology, Weill Cornell Medical College, New York, NY 10065; ^b^School of Electrical and Computer Engineering, Cornell University and Cornell Tech, New York, NY 10044; ^c^Computer Science, Stanford University, Stanford, CA 94305; ^d^Psychiatry and Behavioral Sciences, Stanford Medicine, Stanford, CA 94305

**Keywords:** deep learning, medical image analysis, longitudinal image analysis

## Abstract

Longitudinal imaging data are routinely acquired for health studies and patient monitoring. A central goal in longitudinal studies is tracking relevant change over time. Traditional methods remove nuisance variation with custom pipelines to focus on significant changes. In this work, we present a machine learning–based method that automatically ignores irrelevant changes and extracts the time-varying signal of interest. Our method, called Learning-based Inference of Longitudinal imAge Changes (LILAC), performs a pairwise comparison of longitudinal images in order to make a temporal difference prediction. LILAC employs a convolutional Siamese architecture to extract feature pairs, followed by subtraction and a bias-free fully connected layer to learn meaningful temporal image differences. We first showcase LILAC’s ability to capture key longitudinal changes by simply training it to predict the temporal ordering of images. In our experiments, temporal ordering accuracy exceeded 0.98, and predicted time differences were strongly correlated with actual changes in relevant variables (Pearson Correlation Coefficient *r* = 0.911 with embryo phase change, and r = 0.875 with time interval in wound healing). Next, we trained LILAC to explicitly predict specific targets, such as the change in clinical scores in patients with mild cognitive impairment. LILAC models achieved over a 40% reduction in root mean square error compared to baseline methods. Our empirical results demonstrate that LILAC effectively localizes and quantifies relevant individual-level changes in longitudinal imaging data, offering valuable insights for studying temporal mechanisms or guiding clinical decisions.

## Significance

Longitudinal imaging plays a crucial role for health studies, screening, and monitoring, by enabling the tracking of significant changes over time. Current methods require manual preprocessing with customized pipelines to remove irrelevant variations, such as differences in field of view, noise artifacts, and unintended motion. We present Learning-based Inference of Longitudinal imAge Changes (LILAC), a machine learning method that automatically highlights important temporal changes by comparing image pairs, without depending on custom processing pipelines. Using four real-world datasets, we demonstrate that LILAC accurately predicts temporal ordering and specific targets, like aging intervals and clinical scores, while allowing the localization of relevant changes.

Much of biomedical research and healthcare is about characterizing and responding to relevant changes in data that have been collected over time. These data often include imaging such as mammograms, computed tomography (CT) scans, or MRI. Longitudinal images are commonly acquired in the context of screening (e.g., for breast or lung cancer), population studies (e.g., of aging, neuro-degenerative diseases, and cardiovascular health), and patient monitoring (e.g., to quantify tumor size in response to treatment).

Imaging data provide a snapshot of biological structures, including tissues and organs, at a given moment. Longitudinal images add further value by revealing temporal changes that might be correlated with development, evolution of risk, or progression of disease. Longitudinal design has become an important approach for studying progressive conditions such as cardiovascular disease (1) and neurodegenerative disorders like Alzheimer’s (2). A common approach in these studies is to implement a hand-engineered pipeline to extract image-based measurements (e.g., volume, area, or thickness of an anatomical structure), which are in turn analyzed with statistical methods, such as mixed-effects or spatiotemporal models, to reveal population-level associations (3–8). An alternative strategy constructs image-based longitudinal atlases, instead of using individual-level measurements (9–11). All these prior statistical techniques are sensitive to the quality of image processing steps (e.g., image registration accuracy) and depend on user-defined choices of variables and/or regions of interest.

The promise of deep learning (DL)-based methods like Convolutional Neural Networks (CNNs) (12) or Vision Transformers (13) is the models’ ability to extract features directly from pixel values, instead of relying on hand-crafted feature extraction pipelines. Leveraging temporal convolutions (14), recurrent neural networks (15), or attention modules (16–20), DL can capture the temporal aspects in longitudinal imaging features. DL has been widely applied to improve prognostic outcomes by leveraging temporal similarities in longitudinal data, e.g., refs. 21–23. These studies are not focused on tracking individual-level “temporal changes,” but predicting clinical endpoints. In the present paper, on the other hand, our emphasis is not on prediction but quantifying and mapping significant and relevant changes in longitudinal imaging data.

The major advantage of the longitudinal design over cross-sectional design is that it allows for the disentangling of subject-specific temporal changes from group-level trends. Recently proposed DL-based longitudinal image analysis methods use image comparison to train a model to learn to separate individual disease progression from the estimated timeline (24) and to extract disease-related temporal differences from longitudinal pairs (25). While these methods can improve the characterization of the population-level correlation between independent and dependent variables, they overlook the fundamental value of longitudinal data, where key information is often encoded in the relative changes within individual sequences. Both Couronné (24) and Dong (25) offer summary representations of individual trends through trajectory score plots, yet neither provides an analysis of the relative changes within image sequences.

Recently, self-supervised learning has become increasingly used in longitudinal image analysis. For example, the Longitudinal Self-Supervised Learning (LSSL) model (26) aims to learn a brain representation that disentangles aging from neurodegeneration. Similarly, the Longitudinal Neighborhood Embedding (LNE) method (27) refines brain MRI-derived representations to ensure temporal consistency. This refinement produces smooth trajectories of brain age changes and disease progression. Another self-supervised longitudinal representation learning method for image-to-image architectures demonstrates longitudinal consistency across various segmentation tasks (28). However, these techniques rely on a reconstruction loss, which makes them sensitive to nuisance variations and thus requiring extensive image processing. For instance, Ren et al. (28) applied intersubject affine registration and intrasubject deformable registration to achieve spatiotemporal correspondence, which may inadvertently obscure critical features due to overcorrection. The challenge of reconstruction loss negatively affecting performance when images are not perfectly aligned is well known (29). Because of the reconstruction loss, LSSL (26) and LNE (27) are limited to utilizing low-resolution images (e.g., of size 64 × 64 × 64). Additionally, since these models are designed specifically for studying longitudinal brain MRI images, they cannot be directly applied to other diverse longitudinal imaging datasets, such as microscopy images.

**Fig. 1.**
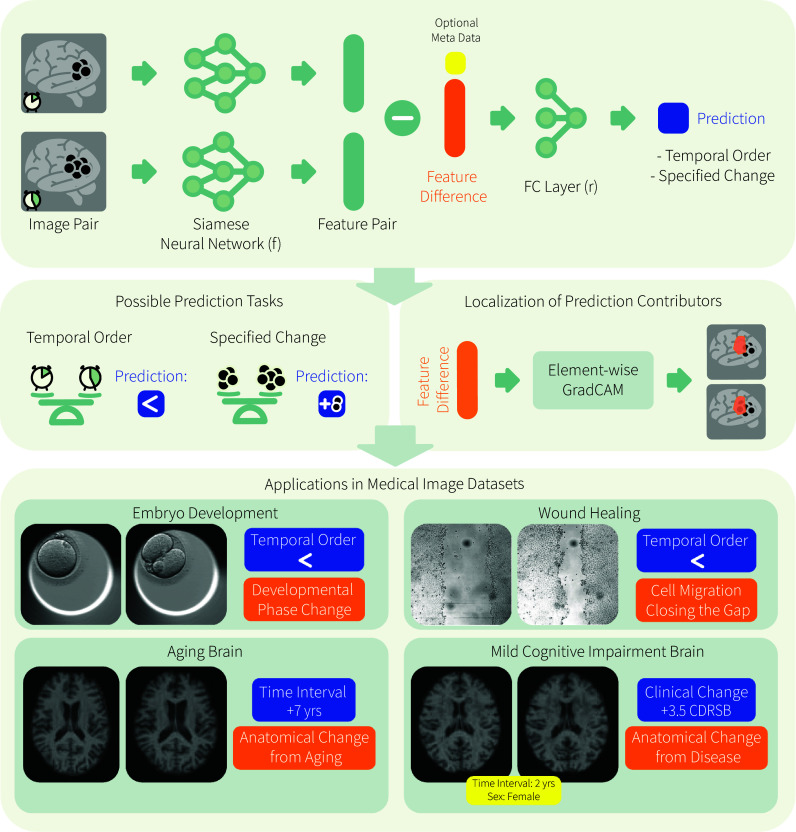
Schematic overview of Learning-based Inference of Longitudinal imAge Changes (LILAC), a learning-based method for inferring longitudinal image changes. (*Top*) Architecture design of LILAC. (*Middle*) Two major prediction tasks are to predict temporal order (LILAC-o) and change of specific value. Examples of predicting specific changes include time intervals (LILAC-t) and cognitive scores with additional variables (LILAC-s). The predictions from each task are utilized for quantitative analysis, while the localization of prediction contributors enables qualitative analysis. (*Bottom*) Real-world applications using medical imaging datasets, with example image pairs demonstrating input for LILAC-based analysis.

In this paper, we present a straightforward, versatile, and effective approach to probing changes in longitudinal images. We refer to our approach as Learning-based Inference of Longitudinal imAge Changes (LILAC, for short). LILAC captures relevant temporal changes in longitudinal images by learning to predict temporal ordering (e.g., without a specific target variable) and changes in time-varying target variables (e.g., clinical scores). To focus the modeling efforts on these changes, we leverage the idea of pairwise image ranking in computer vision that is commonly used for evaluating images and videos (30–33). As depicted in Fig. 1, LILAC extracts features from two images in a longitudinal sequence using the same CNN. The two features are then subtracted and fed to a fully connected (FC) layer without a bias term to make a prediction.

By design, LILAC learns to ignore nuisance factors that are independent of the target variables. This eliminates the need for extensive image processing or costly annotations, making it a practical and efficient tool for longitudinal image analysis. LILAC extends our preliminary PaIRNet work that showed the advantage of pairwise comparisons for inferring temporal difference in longitudinal image data (34). In the present paper, we introduce a supervised approach and demonstrate a way to incorporate optional metadata to disentangle concurrent longitudinal changes. Furthermore, we present a rich set of experimental results.

On a diverse set of application domains, we empirically showcase LILAC via examining its predictive accuracy in quantifying relevant change and via measuring its localization capabilities. In the first set of experiments, we train LILAC to simply temporally order time-lapse images. We refer to this variant as LILAC-o. We view “temporal ordering” as a very general task that is almost always applicable, since time-ordering information is standard in longitudinal studies. In this approach, the model learns to capture time-irreversible nonspecific dynamics, such as those prominent in development, healing, aging, and disease progression, while ignoring changes that are not consistent over time. Our experiments demonstrate that with the temporal ordering task, LILAC-o can reveal insights into processes of embryo development and wound-healing.

Alternatively, LILAC can be trained to predict a specific target variable, such as the time interval between images or change in clinical score. Time-interval prediction can, in theory, capture changes that are not time-irreversible. We refer to this variant as LILAC-t. We showcase that LILAC-t can characterize individual aging patterns using longitudinal MRI scans from a healthy aging group of subjects. Learning to predict specific scores while controlling for the optional metadata allows the model to capture dynamics that are specific to the question of interest. We refer to this variant as LILAC-s. We demonstrate this capability of LILAC-s using longitudinal MRI scans from patients with mild cognitive impairment (MCI). Our results present that LILAC-s can help uncover heterogeneous patterns of temporal changes associated with clinical decline in MCI.

## Empirical Results

### Learning to Order Embryo Time-lapse Images Captures the Development Process.

We first illustrate that by simply training LILAC-o to temporally order images from the same sequence, the model learns to capture important longitudinal dynamics. We utilized a time-lapse imaging dataset of embryo development collected from couples who underwent in vitro fertilization treatments (35) (Fig. 1*Bottom* and *SI Appendix*, Fig. S1). A total of 8,272 images sourced from 704 sets of embryo time-lapse image series were incorporated (11.75±2.44 images per sequence). The images are annotated by experts into 16 developmental phases. This step is time-consuming and requires a visual examination by an expert embryologist.

We trained LILAC-o to predict the temporal ordering of a randomized pair of longitudinal images using binary cross entropy loss. For each image pair, the task is whether the images are in the correct or incorrect order. On the test set, LILAC-o achieved an Area Under the receiver operating characteristic Curve (AUC) of 0.989 (bootstrap-based 95% CI: [0.987, 0.990]) for the binary temporal ordering task. All errors occur in relatively short phase-interval pairs (See *SI Appendix*, Fig. S2 for the AUC across phase intervals). Despite being trained on temporal ordering, the logit output of LILAC-o correlates significantly with the continuous phase change between the two time points [Pearson Correlation Coefficient, or PCC r=0.911 (P<1e−16)]. This result demonstrates that temporal ordering can be a useful task for quantifying relevant change.

**Fig. 2.**
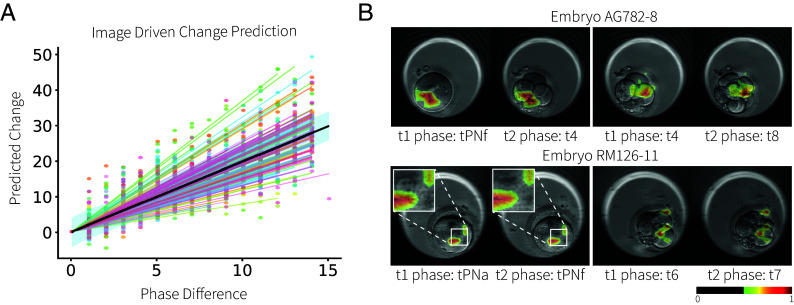
Test results of a LILAC-o network trained for temporal ordering in the embryo development dataset. (*A*) Fitted result of linear mixed effect model between ground-truth phase difference and predicted change. (*B*) Modified Grad-CAM maps overlaid on input images. In the AG782-8 test sample, comparison of phase t4 with tPNf and t8 reveals different regions, correlating with observed cell division. In the RM126-11 test sample, one can appreciate the challenge of comparing images from consecutive phases.

Fig. 2*A* shows all the LILAC-o predicted logits for all image pairs that include the first time point and a later image, for different individuals (indicated in colors). We fit a linear mixed effects model to these samples, allowing for an individual-level random slope. We observe that there is significant variation (P=2.2e−16 assessed with a likelihood ratio test) between individual slopes, which is likely due to differences between the embryo samples.

We implemented a modified Gradient-weighted Class Activation Mapping (Grad-CAM) (36) (see *Materials and Methods* for details) to calculate saliency maps, which reveal the image regions that drive LILAC-o’s predictions. Fig. 2*B* illustrates the modified Grad-CAM results overlaid on input images for four different image pairs. In the example of embryo AG782-8 (first row), we observe that different regions are highlighted when comparing images at different developmental phases. For instance, when comparing the image at the 4-cell (t4) phase with those at the pronuclei disappearance (tPNf) and 8-cell (t8) phases, the model effectively identified changes associated with cell division at distinct locations. In another example of embryo RM126-11 (second row), the phases of pronuclei appearance (tPNa) and disappearance (tPNf) are consecutive phases that undergo subtle temporal transitions. LILAC-o successfully localized the disappearance of pronuclei. Similar to the previous example, the cell division between images from the time of 6-cell (t6) and the time of 7-cell (t7) were also highlighted.

### Characterizing Wound-Healing via Temporally Ordering Time-Lapse Images.

In a second set of experiments, we show that temporal ordering by LILAC-o can capture group-level differences in longitudinal change patterns. We used the wound-healing dataset containing 31 samples with 105.23±49.94 longitudinal time-lapse images per sample (37, 38). This dataset is collected to study the effect of the Met-Hepatocyte Growth Factor/Scatter Factor (HGF/SF) on the mobility of cells by comparing the healing process to an untreated (control) group (Fig. 1*Bottom* and *SI Appendix*, Fig. S3). Traditionally, the healing process is quantified by segmenting the cell (manually or automatically) and the background, and assessing the gap between segmented cells from the two sides (39, 40). The change in the area of this gap is what we refer to as the segmentation-based baseline method.

**Fig. 3.**
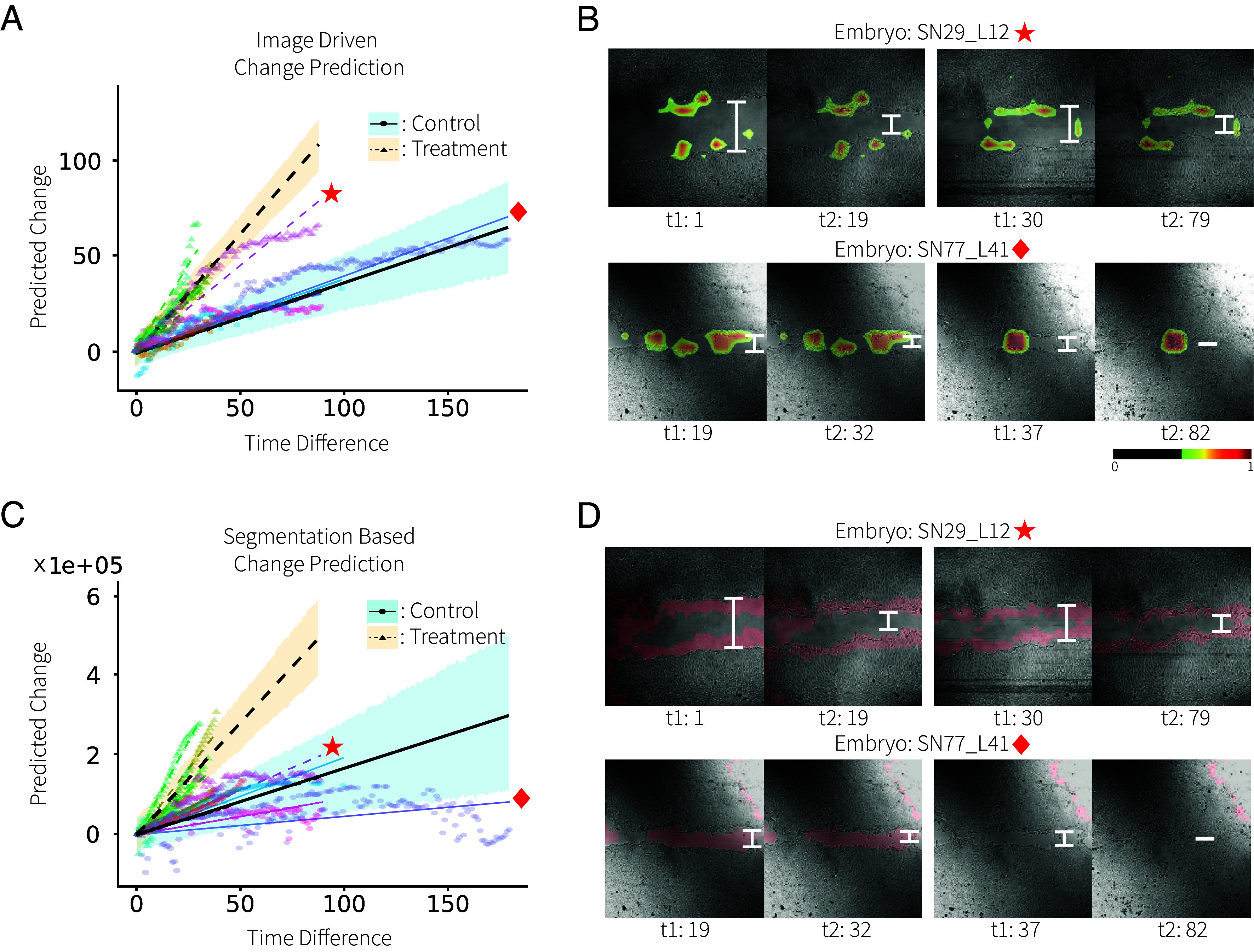
Test results for the wound-healing assay dataset. (*A*) Fitted result of the linear mixed-effects model between ground-truth time difference and predicted change for LILAC-o based prediction. (*B*) shows segmentation-based baseline method’s predictions. LILAC-o predictions better distinguish control and treatment groups, meanwhile, segmentation-based predictions exhibit some important failures (e.g. the star-marked treatment sample). Modified Grad-CAM maps overlaid on input images for LILAC-o: Both SN77_L41 and SN29_L12 highlight where the gap (marked in white) reduces due to cell migration. (*C*) Fitted result of the linear mixed-effects model between ground-truth time difference and predicted change for automatic segmentation-based prediction. In (*D*), both images illustrate cases where the automatic segmentation (red overlay) fails, which is the main cause of the noisy result for the baseline.

We trained and validated LILAC-o in the same way as the embryo dataset. Similar to the previous experiment, validating LILAC-o reveals that it can accurately predict the temporal ordering of images within the same longitudinal sequence [AUC of 0.9908 (bootstrap-based 95% CI: [0.9905, 0.9912])] and the predicted logits significantly correlate with actual time differences [PCC r=0.875 (P<1e−16)]. More critically, the temporal changes estimated by LILAC-o on individual trends from the first time point show a significant difference between treatment and control groups, with the treatment group exhibiting higher slopes indicative of faster healing (Fig. 3*A*). The efficacy of LILAC-o is further emphasized by the larger separation between treatment and control groups, compared to noisy predictions obtained from the change prediction using the conventional segmentation-based baseline method (Fig. 3*C*). For example, the star-marked case (embryo SN29_L12) was from the treatment group but showed a similar slope to the control group in the baseline segmentation results. Also, there are cases in the control group of the baseline segmentation results where the healing process shows near-zero or even a negative slope (e.g., the diamond-marked case embryo SN77_L41 in Fig. 3), which is not expected. These implausible trends in Fig. 3*C* are likely due to the poor quality of the automatic segmentation in SN29_L12 and SN77_L41 (Fig. 3*D*). On the other hand, LILAC-o is successful at localizing the relevant changes, as evidenced by how the modified Grad-CAM maps highlight the cells moving from the two ends of the wound gap (Fig. 3*B*).

### Characterizing Individualized Brain Aging Through Predicting Time Interval.

Next, we demonstrate LILAC-t’s capability to probe longitudinal change with another example, showing that the model can effectively capture individualized temporal changes by learning to predict the time interval between the images, instead of just temporal ordering. We utilized 754 3D T1-weighted (T1W) MRIs of 272 healthy subjects (2.77±0.96 images per subject, subjects were 64.34±8.53 y old at their first visit, and the longest temporal interval was 4.72±2.30 y) from OASIS-3 (41) (Fig. 1*Bottom* and *SI Appendix*, Fig. S4). Different from the temporal ordering task, we trained LILAC-t with the Mean Squared Error (MSE) loss to predict the continuous time difference between the input image pair.

The predicted time difference by LILAC-t has a root MSE (RMSE) of 1.825 y, which was significantly lower than a naive prediction baseline that always predicts 0 as the time difference (RMSE of 3.49 y). The time difference predicted by LILAC-t was also more accurate than a single image regression baseline (SIRB) (RMSE of 4.973 y), which predicts the age of a given image and calculates the difference in predicted age between a pair of longitudinal images. Although direct comparisons may be challenging, it is worth noting that LILAC-t achieved a Mean Absolute Error (MAE) of 1.087 y for age difference prediction, comparable to the typical MAE of 2.14 to 4.16 reported by state-of-the-art methods for age prediction from T1W MRI (42–45). Similar to the results from temporal ordering tasks, the predicted time difference is highly correlated [PCC r=0.853 (P<1e−16)] with the ground-truth time difference and statistically significant variation (P=6.303e−6) was observed between individual slopes (*SI Appendix*, Fig. S5).

**Fig. 4.**
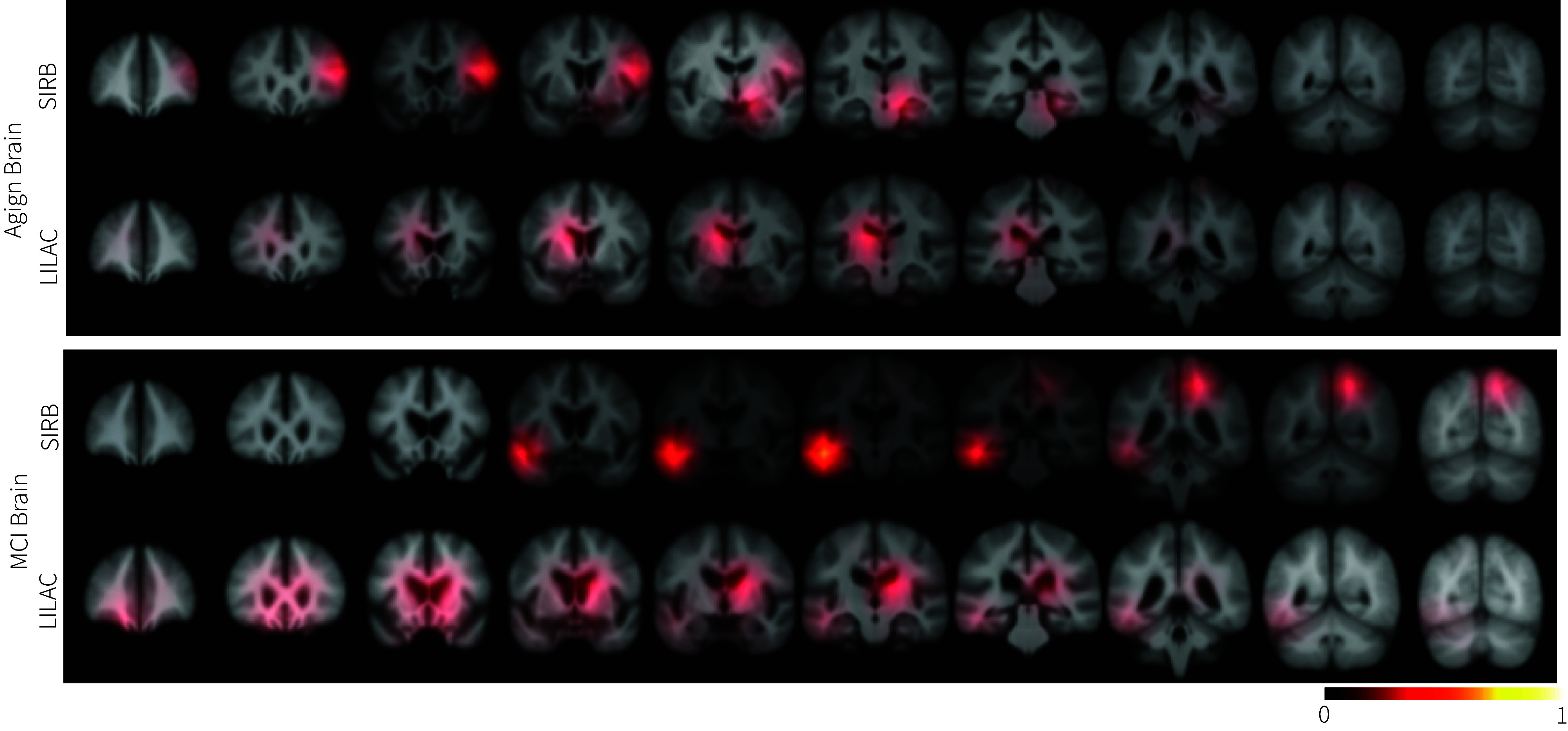
Population-average modified Grad-CAM peaks computed over the test set from the maximum time difference pair for each subject. The heatmaps are overlaid on the averaged image of the whole test set of each dataset. The more distributed nature of LILAC-t implies it is better at capturing individual variability. On the other hand, a Single Image Regression Baseline (SIRB) produces more concentrated Grad-CAM saliency map peaks.

**Fig. 5.**
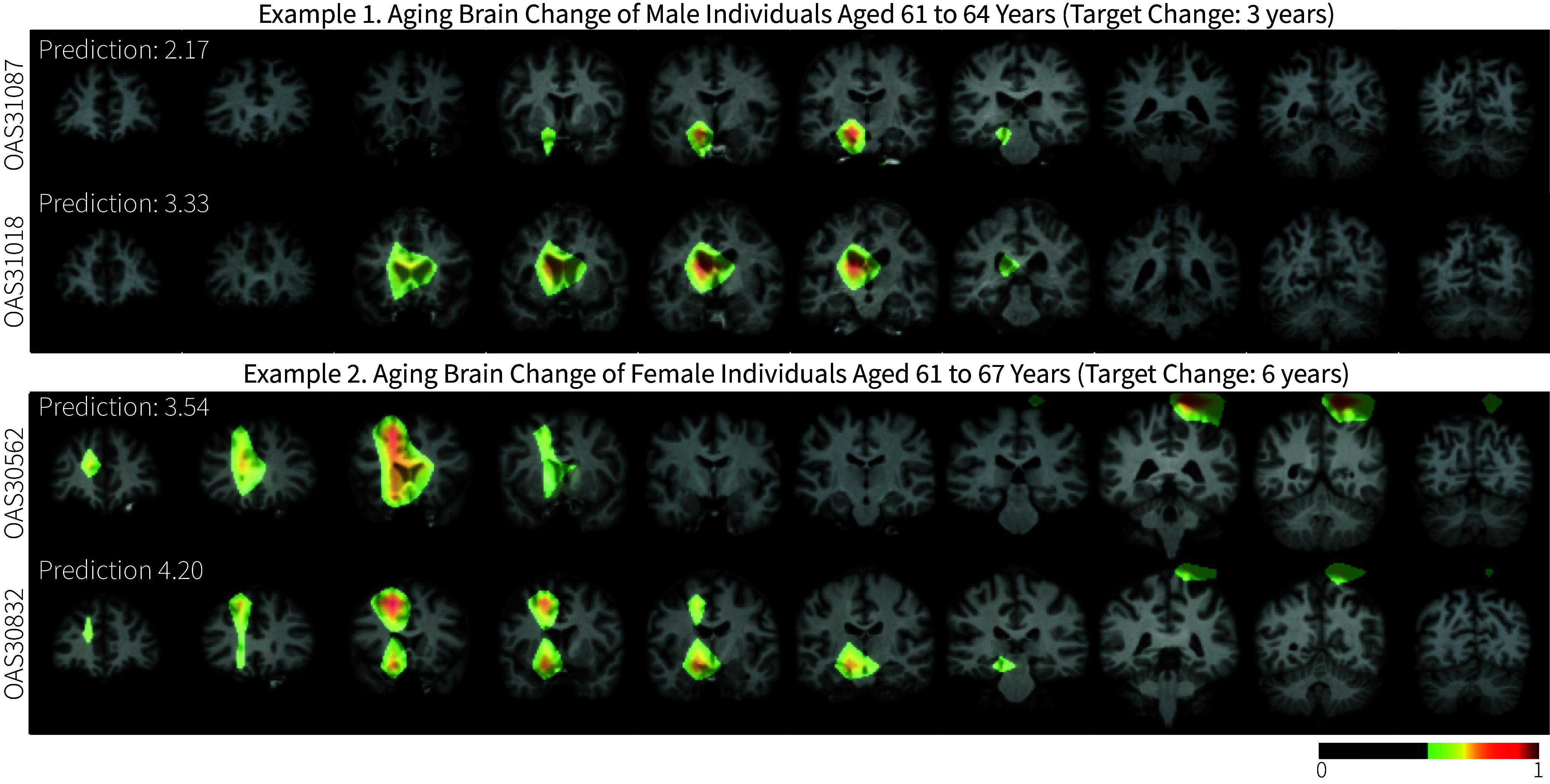
Modified Grad-CAM results overlaid on input images, illustrating distinct patterns of change across test subjects. Trained for time interval (i.e., age difference) prediction in an aging brain dataset, LILAC-t captures individual variations in longitudinal change even between pairs of subjects of the same age and sex. The images displayed are later time points (t2) of the pairs.

While the aging brain exhibits common alterations, the pattern and pace of change can vary among subjects. Fig. 4 illustrates the distribution of the peak locations of Grad-CAM saliency maps, with LILAC-t’s being more sparse than SIRB. The distribution is obtained by binarizing the heatmaps, with values of 1 representing peak locations (on the (8×8×8) grid, then up-sampled to full-resolution), and averaging them across all test cases. To ensure equal consideration of subjects regardless of the number of timepoints, all experiments use the maximum time difference pair for each subject (i.e., the first and last scans). These peak distributions suggest LILAC-t is better at capturing individual variability in aging. The peak distribution from LILAC-t is consistent with the findings from the analysis of volumetric changes in prespecific ROIs (*SI Appendix*, Fig. S6). This distribution reflects heterogeneity across subjects, and highlights regions near the left ventricle and left white matter, exhibiting a lateralized pattern. Fig. 5 further shows the saliency maps associated with two representative pairs of subjects. Even though each pair of subjects had the same sex and chronological age, LILAC-t reveals distinctive change patterns, showcasing its capability to capture individual nuances. Meanwhile, the saliency maps of the corresponding image pairs for SIRB show similar patterns (*SI Appendix*, Fig. S7).

### Probing Specific Changes via Learning to Predict Variation in Clinical Scores.

In many real-world scenarios, there can be multiple simultaneous temporal processes. For example, aging and pathological neurodegeneration (as in Alzheimer’s) often co-occur. In the following set-up, we demonstrate how LILAC-s can be adapted to disentangle different processes, and infer specific longitudinal changes. We trained LILAC-s to predict the change in the clinical Dementia Rating Scale Sum of Boxes (CDRSB) between an image pair with MSE loss. To disentangle changes associated with aging and dementia-associated neurodegeneration, we provided the time interval between the images, and sex indicator (1, if male) multiplied with the time interval, as additional input variables to LILAC-s. This way, the model only learns to extract the signal that is associated with CDRSB change, above and beyond aging and sex-specific aging. For the SIRB, we provided age and sex as additional input variables and the model predicts concurrent CDRSB. We used 3,616 T1W images of 749 MCI subjects with associated CDRSB scores from the Alzheimer’s Disease Neuroimaging Initiative (ADNI) study (Fig. 1*Bottom* and *SI Appendix*, Fig. S8). The subjects have 4.83±1.79 images and were 73.38±7.42 y old at their first visit. The average CDRSB at the first visit was 1.22±0.86, and the average CDRSB difference for the longest temporal interval was 2.36±2.57. LILAC-s predicted the CDRSB difference between time points with an RMSE of 1.415, significantly better than the naive baseline of predicting 0 (RMSE of 2.482) and SIRB (RMSE of 1.755).

Similar to the aging brain result, the sparsity of the Grad-CAM saliency maps’ peak locations in Fig. 4 illustrates the variability in localization across subjects, whereas the SIRB predominantly localizes the temporal lobe in the majority of images (See *SI Appendix*, Fig. S9 for subject-wise examples).

**Fig. 6.**
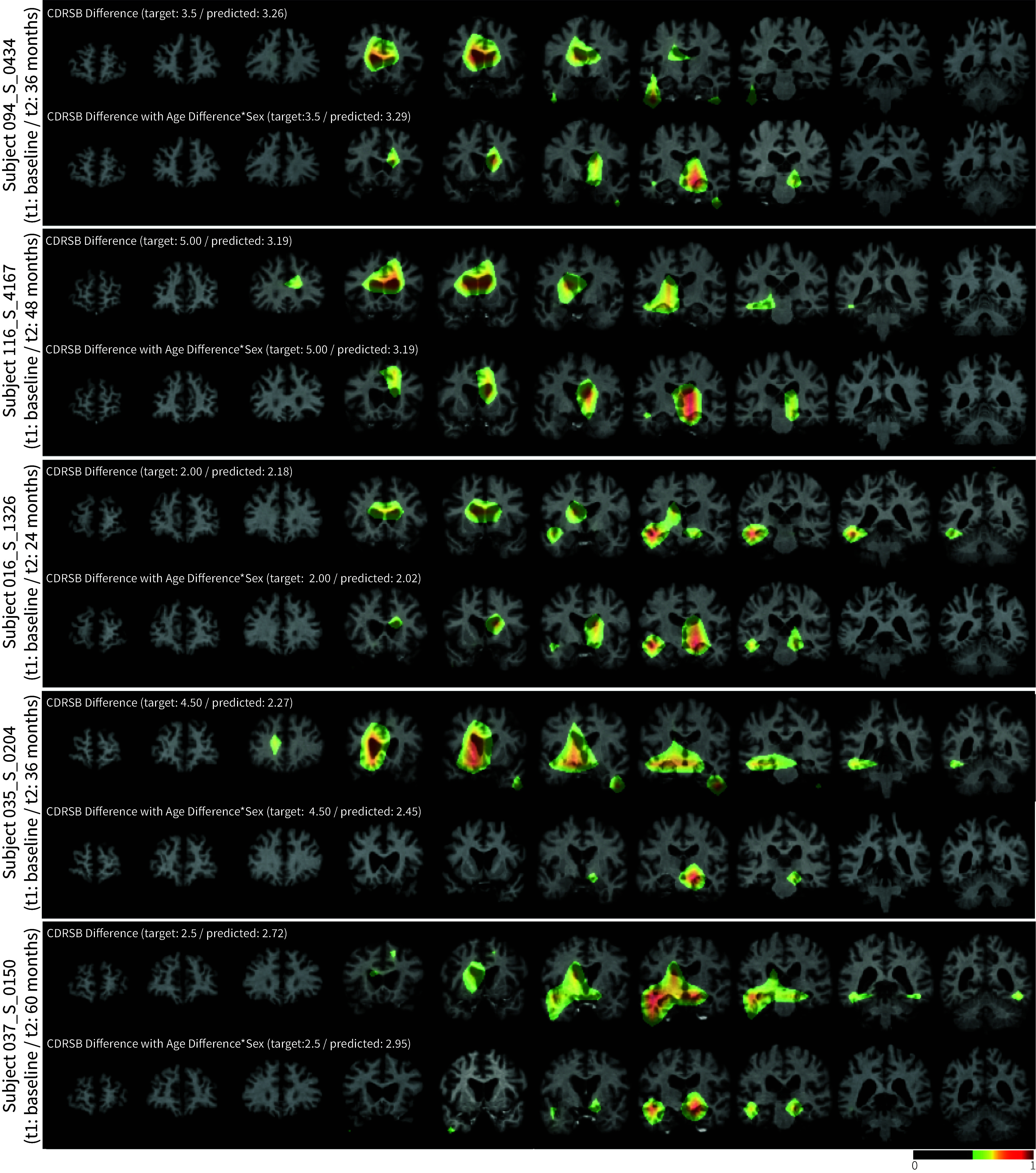
Modified Grad-CAM results overlaid on input pairs from representative test subjects, where LILAC is trained to predict the change in clinical Dementia Rating Scale Sum of Boxes (CDRSB) (*Top*), while accounting for age difference and sex as additional variables (*Bottom*). The different patterns highlight the importance of controlling for metavariables in interpreting localization results.

Fig. 6 shows Grad-CAM maps for two different LILAC-s models, trained on the same MCI subjects to predict CDRSB change, with and without controlling for aging and sex. While both models resulted in high PCC between predicted and ground-truth change (*SI Appendix*, Table S1), we observe important differences in the saliency maps. This suggest that the models make their predictions using different parts of the images. For instance, for subjects 094_S_0434, 116_S_4167, and 016_S_1326, to predict the CDRSB change without controlling for aging and sex, the ventricles and temporal lobe are highlighted. When controlling for aging and sex, the temporal lobe is the primary driver of the prediction. In the case of subjects 035_S_0204 and 037_S_0150, we also observe different regions being highlighted depending on whether we control for aging and sex or not. Further heatmaps for predicting CDRSB while controlling only for age can be found in *SI Appendix*, Fig. S10.

## Discussion

In this paper, we present a learning-based method for longitudinal image analysis called LILAC. The primary goal of longitudinal image analysis is to localize and quantify relevant temporal changes. This may include correlating the imaging findings with lab test results, patient demographics, and clinical symptoms. Traditional longitudinal image analysis methods employ statistical models, like mixed-effects and spatiotemporal regression models, that can handle repeated measures. These techniques, however, are often limited by the specified model and the image processing steps, and they often only produce population-level insights with limited characterization of individual-level dynamics. Deep learning methods promise to capture complex and nonlinear individual-level patterns present in images, without relying on image processing.

As we show in this paper, LILAC can quantify and localize the relevant changes by comparing longitudinal image pairs directly on pixel-level data and without heavy preprocessing or hand-crafted features.

The longitudinal design offers the ability to better track and characterize individual-level temporal dynamics, and LILAC provides a straightforward way to do that. Our experiments show that LILAC outperforms a strong baseline that was trained directly to predict the target variable from individual images. LILAC quantifies and localizes individual differences in aging brains, as shown in the slope variability of individual-level predictions (*SI Appendix*, Fig. S5) and the saliency map results (Figs. 4 and 5). In contrast, the patterns of SIRB localization are similar across individuals as shown in *SI Appendix*, Fig. S7.

We demonstrate that temporal ordering (LILAC-o) can be a simple, yet effective task for capturing time-irreversible patterns. Our results from the embryo and wound-healing assay experiments show how temporal ordering can localize and quantify relevant change. Temporal ordering can even reveal important group-level differences hidden in longitudinal data, as our results from the case–control analysis of the wound healing dataset revealed (Fig. 3*A*). When compared to the baseline method that relies on a custom-built automatic segmentation step, LILAC-o produces better predictions without the need for customization. Furthermore, the automatic segmentation approach struggles to differentiate between the cell and background area in certain low-quality or low-contrast images (Fig. 3*D*). This result underscores the benefit of the proposed approach, where nuisance factors are ignored automatically through learning. Although there exist other methods for predicting and evaluating embryo development and the wound healing process, such as direct stage prediction and implantation outcomes for embryos (46), as well as quantification of skin innervation (47), our goal is not to claim superiority over these methods but to demonstrate that the LILAC framework can be effectively applied to a variety of longitudinal change scenarios. Additionally, direct stage prediction or wound gap prediction often requires a manual annotation step, which is both expensive and not always feasible. If the goal is to capture changes without the need for labels, LILAC-o provides an effective alternative, as demonstrated in both the embryo and wound-healing results.

We also presented another flavor of LILAC, LILAC-s, that allows the method to capture specific changes that are not necessarily time-irreversible. In addition, LILAC-s discerns nuanced changes by allowing the user to control for relevant confounding variables. We showcased this idea via characterizing brain changes in patients with MCI, associated with decline toward dementia (Fig. 6 and *SI Appendix*, Fig. S10). The LILAC-s model, used in the MCI brain experiments, is trained to predict changes in CDRSB score, which can exhibit nonmonotonic trajectories for some patients.

Our experiments demonstrated how localized changes driving the predictions can provide some interesting insights. The localized changes in the embryo experiments, for instance, highlight regions of cell division at different developmental phases, whereas in the wound-healing assay experiments, the saliency maps focus on the areas where cell migration occurs. In brain images, LILAC reveals individualized patterns in changes associated with aging and Alzheimer’s disease, which are known to be heterogeneous processes (48, 49). For example, LILAC’s saliency maps show that subjects of the same sex and age can show different patterns of brain changes associated with aging (Fig. 5). We observe similar individual variations in Alzheimer’s associated brain alterations (Fig. 6). The various asymmetric patterns may indicate inherent differences in how two hemispheres respond to aging. For instance, the right hemisphere tends to exhibit more rapid structural decline in areas associated with spatial and emotional processing, whereas the left hemisphere generally experiences greater declines in language and verbal abilities (50, 51).

We utilized the Neural Preprocessing (NPP) tool (52) to preprocess brain images for LILAC-s experiments, enabling the networks to focus on brain regions. However, LILAC does not require preprocessing. To demonstrate this, we ran additional experiments for aging brain with two configurations: 1) raw images without any type of preprocessing, and 2) NPP-processed images excluding MNI registration as ablation. To handle the broader diversity of raw images, we added a convolutional block and applied extensive data augmentation, achieving comparable RMSE and MAE values for aging prediction (*SI Appendix*, Table S2). Grad-CAM visualizations (*SI Appendix*, Fig. S11) showed that raw image heatmaps highlighted broader regions, overlapping with those highlighted in the Grad-CAMs of the preprocessed images. The smoother nature of these maps is due to lower spatial resolution introduced by the added convolutional block. Some results also highlighted the neck region, suggesting potential age-related cervical spine features, as noted in previous studies (53–55). Since the OASIS and ADNI studies aim to investigate the brain, preprocessing steps like skull stripping and linear registration can improve efficiency and help focus on brain-specific changes, as supported by *SI Appendix*, Table S2.

We note some failure cases for each task. In the embryo development experiment, LILAC-o achieved an AUC of over 0.98 for predicting temporal ordering but struggled with images that were close in phase. The most common failed cases were pairs of images containing 9+ cells and the morula stage. The morula stage, which typically spans from 12 or more cells to the onset of blastulation, poses a challenge for accurate prediction due to the indistinct cell boundaries during this phase, as shown in *SI Appendix*, Fig. S14. Similar to embryo development, the failure cases in wound healing occurred with image pairs that were close in time. Half of the failure cases involved pairs that were within 6 time points of each other, which can be challenging, as depicted in *SI Appendix*, Fig. S14. For regression tasks, we used the average MSE plus two SDs as the threshold to identify failure cases. In the aging prediction experiment, half of the failure cases came from the same subject, where one of the timepoint images looked distinctly different (*SI Appendix*, Fig. S14). In the MCI experiments, more than half of the failure cases involved image pairs with a large CDRSB difference, 6 or higher. Pairs with an extraordinarily large target value difference can be more challenging to predict accurately due to fewer data points. Considering that the CDRSB range is between 0 and 18, this large difference may indicate cases with accelerated progression. For all tasks, the localization of image difference was done using Grad-CAM saliency maps. Although Grad-CAM is beneficial for visualizing the models’ focus, this approach has limitations when used for quantitative measurements. They offer low-resolution maps that are often biased toward dominant features and their reliability is contingent upon the accuracy of the model’s predictions. To mitigate this, we only considered the pairs with a high explainability score, τ above 0.7, which is calculated similarly to the $r^{2}$ score.

Several limitations should be acknowledged. First, as in any data-driven approach, LILAC’s performance can be sensitive to the quality and quantity of data, including any biases that might be present, and will depend on computational resources available. Second, while we found the simple CNN architecture for feature extraction to be effective, more complex architectural designs, such as transformer-based networks (56), could potentially improve LILAC’s ability to capture fine-grained changes between the images and extend beyond comparing just two images. The improved architecture could also help reduce the spatial mismatch between the heatmap and the original image by incorporating higher-resolution feature maps during the up-scaling process. Additionally, it may refine the model’s focus, ensuring that attention is concentrated more precisely within brain regions and minimizing influence from nonbrain areas. Third, in many real-world scenarios, the relevant change is not simply captured by a single quantitative variable (e.g., CDRSB). Instead, images are often accompanied by a rich set of other data types, such as test results and clinical reports. These additional data are likely critical for extracting the relevant information from the images. For example, BioViL-T showed the opportunity of using report and image pairs for learning temporal structure (57). Last, we did not explore the potential utility of LILAC features and predicted scores computed on single images. *SI Appendix*, Fig. S12 plots single-image predictions from LILAC-o, which was trained on temporal ordering. These results suggest that single-image predicted values can capture individual variability and group-wise differences.

Addressing these limitations will not only extend the capabilities of LILAC but also broaden its application to a wider range of longitudinal datasets and clinical scenarios. Additionally, future efforts will focus on applying LILAC to datasets with higher variability and different medical imaging domains. These efforts will help refine and enhance LILAC’s utility, ensuring its adaptability to a broader range of longitudinal data challenges.

We believe that LILAC holds significant promise for both clinical workflows and research, as it offers an effective and easy way to track relevant change over time. There are scenarios where dynamic changes are prioritized over static assessments, such as in cancer therapy response, changes in heart function, and orthopedic recovery. LILAC provides a streamlined approach that allows for easy adaptation to various types of longitudinal image datasets, minimizing the complexity and customization often associated with other methods. This flexibility makes LILAC accessible to a wide range of applications, while its simplicity ensures that it can be implemented without the need for extensive customization. It can be applied off-the-shelf to any longitudinal imaging dataset. The user needs to specify the target task (temporal ordering, time interval prediction, or change prediction) and provide the metadata for any confounds to control for. Capturing longitudinal changes while considering individual trends can facilitate more timely and accurate clinical decisions, particularly regarding disease progression and treatment response, ultimately improving patient outcomes. We hope this paper will encourage further study of changes across diverse application domains. Our approach is not limited to clinical applications; it also has potential uses in other fields that track dynamic processes, such as molecular imaging.

## Materials and Methods

### Learning-Based Assessment and Identification of Longitudinal Image Change (LILAC).

A prevalent framework in pairwise image comparison employs a Siamese architecture (30–33) that has a shared feature extraction backbone, followed by a classifier to rank and classify pairs of input images. Similarly, LILAC adopts the design principles of ranking networks (58, 59) and is largely based on PaIRNet (34), originally developed for longitudinal medical image analysis and validated on 2D datasets. The present paper explored LILAC’s ability to assess and identify changes in longitudinal 2D and 3D images at the individual level, correlating with specific targets such as clinical scores, and controlling for confounders like aging.

The architectural design of LILAC is illustrated in Fig. 1*A*. LILAC takes as input two longitudinal images $\left(I_{t1},I_{t2}\right)$ from the same subject, with the convolutional feature extractor network f shared between them, following a Siamese architecture design. The flattened feature vectors, $f(I_{t1})$ and $f(I_{t2})$, are then subtracted and passed through a FC linear layer without a bias term. The resulting output, $r(I_{t1},I_{t2})$, is obtained by taking the dot product of the weight vector w with the difference between feature vectors $f(I_{t1})$ and $f(I_{t2})$. Here, w represents the weights of the bias-free FC layer, and ^*⊤*^ denotes vector transpose. When optional metadata (i.e., nonimaging variables such as subject age) is used, the resulting output can be written as $r\left(I_{t1},I_{t2}\right)=w^{⊤}\left(\left(f\left(I_{t1}\right)\cdot M_{t1}\right)-\left(f\left(I_{t2}\right)\right)\cdot M_{t2}\right)$, where · denotes concatenation and $\left(M_{t1},M_{t2}\right)$ are two metadata from the same subject. For LILAC-o and temporal order prediction (binary classification) tasks, this output is then fed into a sigmoid unit σ, producing a probability. Note that LILAC-o respects the three ranking network properties: reflexivity, antisymmetry, and transitivity (see *SI Appendix* for further details on the theoretical properties).

#### Implementation details.

The feature extraction CNN model consists of four convolutional blocks, each containing a convolution layer, BatchNorm, leaky ReLU activation, and a pooling layer, arranged in sequence. We opted for a lightweight architecture with just 106 K parameters for processing 3D images, instead of using a larger model like a 3D ResNet50, which has 48 million parameters. This decision was driven by the minimal performance difference we observed with larger models in our experiments and our focus on computational efficiency. We experimented with two different spatial pooling methods; average and max pooling. *SI Appendix*, Fig. S13 illustrates the architectural details for 2D and 3D inputs. $R^{2}$, also known as the coefficient of determination, measures the certainty of predictions by showing goodness-of-fit in regression tasks. We used both $R^{2}$ and loss on the validation data to perform model selection for each dataset. Since the feature extraction CNN with average pooling performed the best for all experiments (*SI Appendix*, Table S3), we only reported the average pooling results. We reported the results for SIRB methods using max pooling, as these outperformed the models with average pooling.

In the regression tasks, the LILAC models were trained using MSE loss to predict changes in a clinically significant variable y, such as chronological age or cognitive scores. On the other hand, for LILAC-o (i.e., the temporal ordering task), we employed Cross-Entropy (CE) loss. We utilized Adam optimizer with default parameters and learning rate of $10^{-3}$ (60). The choices of hyperparameters for each experiment are demonstrated in *SI Appendix*, Table S4. Early stopping was used during training, stopping when the validation loss did not decrease for 10 consecutive epochs. The validation set was used to choose the best model. Batches were randomly selected for training in all experiments, except for the MCI brain dataset. For this dataset, the network was initially trained on simpler tasks for the first 10 epochs, given the optimization challenges. During this phase, we began with image pairs separated by more than 50 mo and gradually included more pairs every 2 epochs.

All experiments were conducted using NVIDIA TITAN Xp with 12 GB memory. We implemented our models using PyTorch (ver. 1.10.1) with python 3.9, CUDA version 11.3, and cuDNN version 8.2. The source code of LILAC is available on GitHub (https://github.com/heejong-kim/LILAC).

#### Dataset.

All datasets were randomly split into training, validation, and test sets with proportions roughly equal to of 60%, 20%, and 20%. The splits were performed on a subject-wise basis to avoid data leakage. The details of the total number of images and pairs for each experiment and split are available in *SI Appendix*, Table S5.

(1) Embryo Development Dataset: A time-lapse embryo dataset is publicly available, collected from couples who underwent intracytoplasmic sperm injection (ICSI) cycles (35). The time-lapse sequences are manually annotated by an embryologist for 16 cellular events: tPB2, tPNa, tPNf, t2, t3, t4, t5, t6, t7, t8, t9+, tM, tSB, tB, tEB, and tHB. Each embryo contains an average of 97.13±1.673 images and 11.75±2.44 phases per sequence. We utilized one image per phase in this paper. A total of 8,272 images were used. Representative examples of the embryo dataset can be found in *SI Appendix*, Fig. S1.
(2) Wound-healing Assay Dataset: A wound-healing assay, also known as a scratch assay, is an approach to study the cell migration/wound-healing process. The wound-healing dataset used in this paper is a publicly available dataset collected to examine the effect of HGF/SF on the mobility of cells by comparing the process to a control group left untreated (*SI Appendix*, Fig. S3) (37, 38, 61). The dataset consists of 3,262 time-lapse images in total. For the segmentation-based analysis, we only used images with segmentation labels. The segmentation labels, which are automatically generated based on the algorithm proposed in ref. 62, are provided with the data release.
(3) Aging Brain Dataset: The images used for brain aging experiments were obtained from OASIS-3, a longitudinal neuroimaging study focused on normal aging and Alzheimer’s Disease. We selected healthy cohorts with a CDRSB score of zero, totaling 754 images. We used the NPP tool (52) to convert raw brain images to skull-stripped, intensity-normalized images and register those to the MNI image space.
(4) Mild Cognitive Impairment Brain Dataset: Data used in the preparation of this article were obtained from the ADNI database (https://adni.loni.usc.edu). The ADNI was launched in 2003 as a public–private partnership, led by Principal Investigator Michael W. Weiner, MD. The primary goal of ADNI has been to test whether serial MRI, positron emission tomography, other biological markers, and clinical and neuropsychological assessment can be combined to measure the progression of MCI and early Alzheimer’s disease (AD). We only used the subjects that were recruited in the MCI group, totaling 4,006 images. The images were processed using the NPP tool (52), as described above.

### Model Evaluation.

To assess the performance of the trained LILAC model on each dataset, we conducted comparisons between the model’s predictions and those of a naive baseline. In binary classification tasks, the naive prediction baseline is selecting the majority class across all instances. Thus, in temporal-ordering tasks where the number of positive and negative pairs is equal, the naive prediction defaults to 0.5. We used bootstrapping to estimate the variability of the AUC by sampling the test set with replacement 1,000 times. This allows us to assess the stability and CIs of the AUC. For regression tasks, such as time interval or clinical score change predictions, we utilized the mean of the target variable (which is 0) for all instances, against which we evaluated using the L2 loss.

### Quantification of Image-Derived Change.

To quantify the prediction performance, we calculated Pearson correlation coefficients (63) between ground-truth change in target variable and predicted change in regression tasks or logit (presigmoid) values in temporal ordering tasks. We exclusively used temporally ordered image pairs in our correlation analyses. For further evaluation, we employed a linear mixed effect (LME) model-based analysis (4), where we considered the temporal changes starting from the initial time point value of each subject. Since the (predicted and observed) change at the initial time point is zero, we did not include intercepts in the LME analysis and only considered subject-level random effects for the slope. The statistical significance of individual variability was assessed by performing a likelihood ratio test, comparing a model with random effects to a reduced model without random effects. In the wound-healing assay experiment, we tested for the hypothesis that the speed of healing was different between treatment and control groups. In the LME model, we included the group (treatment/control) as an additional fixed effect variable. Similar to assessing individual variability, the significance of the group effect was evaluated by comparing models with and without the group term. The LME models were fit using maximum likelihood estimation. The mathematical representation of models can be found in *SI Appendix*, *Method*.

### Localization of Image-Derived Change.

The Gradient-weighted Class Activation Mapping (Grad-CAM) (36) is a popular method to probe image classifiers trained only on image-level labels to get insights into which parts of an image the model is focusing on for a prediction. In the original Grad-CAM method, the importance of feature maps is computed by taking the global average pooling of the gradient-weighted activations at the last layer. The Grad-CAM saliency map is calculated by weighting the activation maps of the last convolutional layer by the importance scores. The ReLU function is applied to ensure positive contributions are retained.

Building on this idea, we modified Grad-CAM to localize objects of interest that the models used to make predictions. Since we used flattening instead of global average pooling, we calculated the importance scores element-wise. Each gradient-weighted activation at the last layer is multiplied element-wise with the activation maps. Because reliability of the Grad-CAM result depends on prediction quality, reported saliency maps are from the cases that exhibit explainability score above 0.7. The score was quantified as $\tau_{i}=1-{\left(y_{i}-{\hat{y}}_{i}\right)}^{2}/{\left(y_{i}-\overset{¯}{y}\right)}^{2}$, with $y_{i}$ as the ground-truth values, $\hat{y_{i}}$ as the predicted values, and $\overset{¯}{y}$ as the average of the ground-truth values in the training data. This score is similar to the $r^{2}$ score in regression tasks but without summation over data. For binary prediction tasks, we treated this τ as a pseudoscore similar to Efron’s pseudo $r^{2}$ (64).

### Ethical Considerations and Data Use.

This study involves the analysis of deidentified, publicly available data from the ADNI and OASIS-3: Longitudinal Multimodal Neuroimaging databases. Given that the studies do not include any direct interaction with human subjects or access to identifiable private information, it falls under the category of ‘Not Human Subject Research’. The research was conducted in full compliance with all applicable ethical standards and with respect to the privacy and confidentiality of the data.

## Supplementary Material

Appendix 01 (PDF)

Dataset S01 (DOCX)

## Data Availability

Previously published data were used for this work: For embryo, a time-lapse embryo dataset is publicly available, collected from couples who underwent ICSI cycles (35). The wound-healing dataset in this paper is a publicly available dataset collected to examine the effect of HGF/SF on the mobility of cells by comparing the process to a control group left untreated (37, 38).
