# Supplementary material for: Learning-based inference of longitudinal image changes: Applications in embryo development, wound healing, and aging brain
Source: Proc Natl Acad Sci U S A. 2025 Feb 20;122(8):e2411492122. doi: 10.1073/pnas.2411492122 (PMC11873959; doi:10.1073/pnas.2411492122)
Supplement: Supplementary file 1 — Appendix 01 (PDF) [file pnas.2411492122.sapp.pdf]

Supporting Information for

Learning-based Inference of Longitudinal Image Changes: Applications in Embryo Development, Wound Healing, and Aging Brain

Heejong Kim, Batuhan K. Karaman, Qingyu Zhao, Alan Q. Wang, Mert R. Sabuncu

Heejong Kim
E-mail: hek4004@med.cornell.edu

This PDF file includes:

Supporting text
Figs. S1 to S14
Tables S1 to S5
SI References

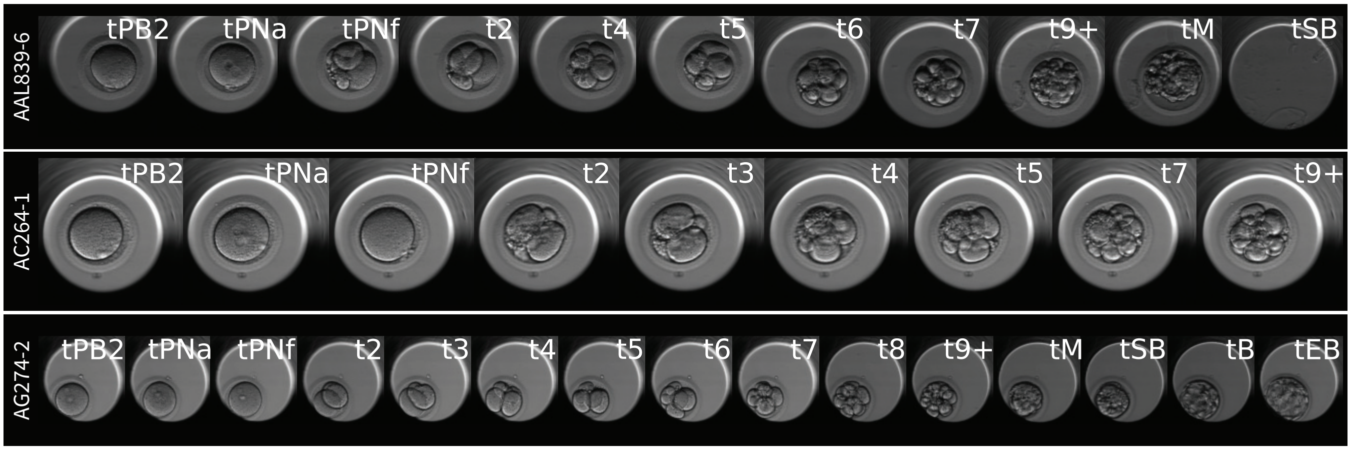

Fig. S1. Representative examples of embryo dataset. Phase labels are marked in the top right corner of each image.

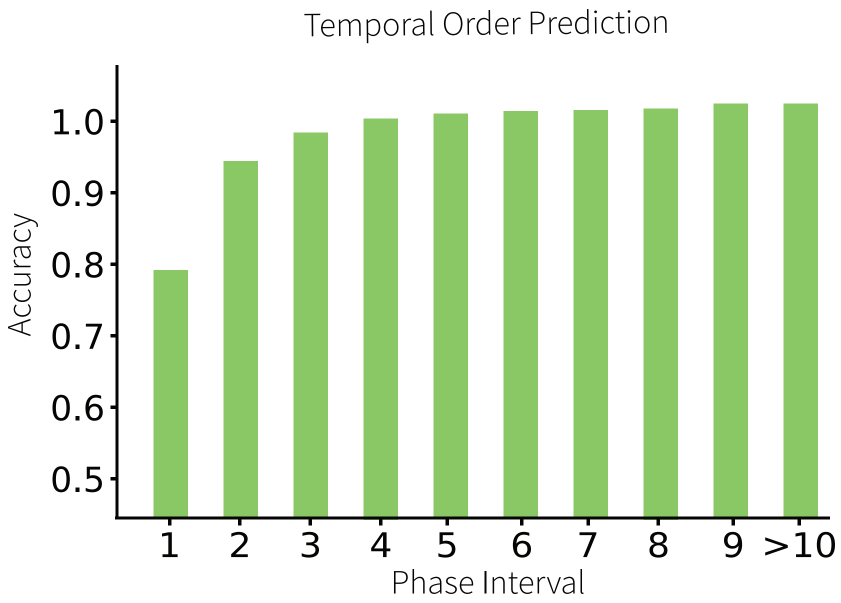

Fig. S2. Area Under the Receiver Operating Characteristic Curve (AUC) for temporal ordering prediction for embryo pairs with different time intervals.

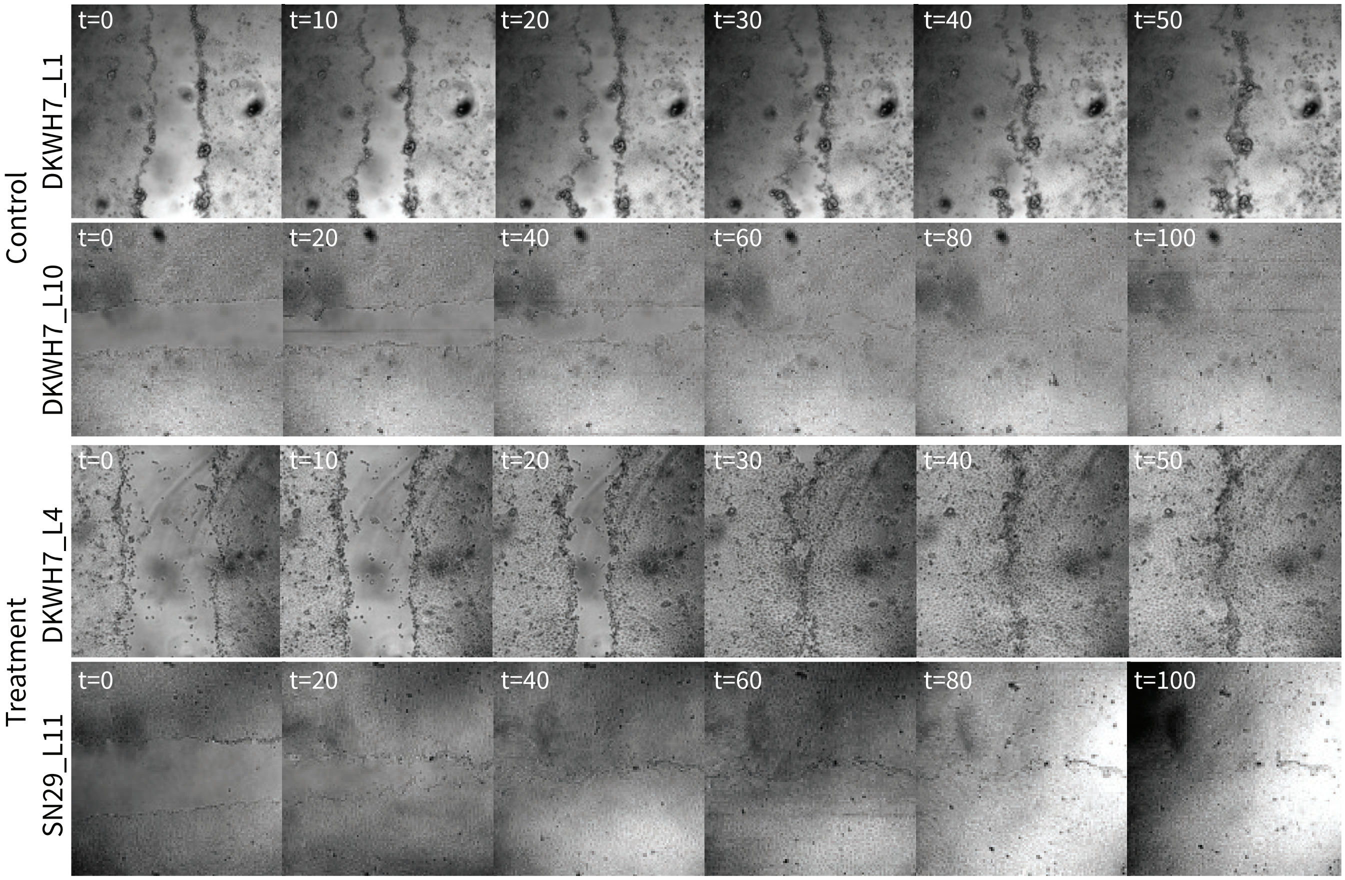

Fig. S3. Representative examples from the wound-healing assay dataset. Time points are indicated in the top left corner of each image. Each row illustrates six images from the sample's longitudinal sequence.

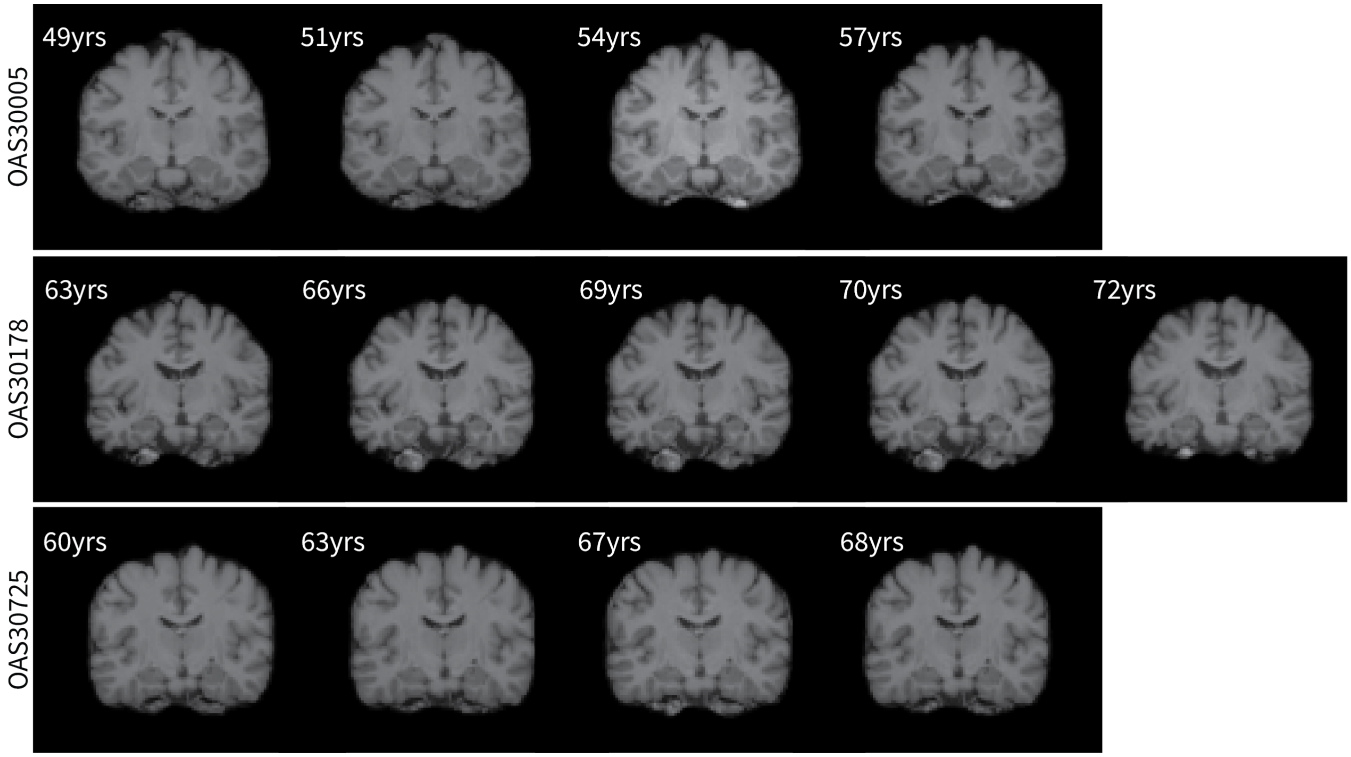

Fig. S4. Representative examples from the aging brain dataset, with the subject's age at scan indicated in the top right corner.

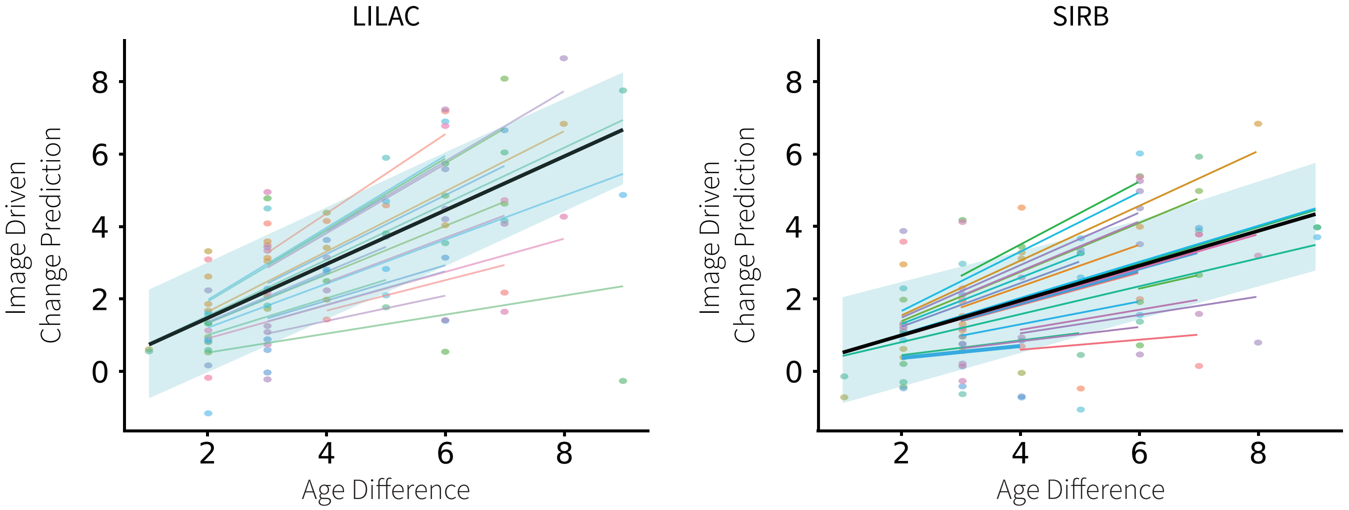

Fig. S5. Fitted result of Linear Mixed Effect Model between ground-truth age difference and predicted change. LILAC is trained to predict time difference. For the single image regression baseline (SIRB), the same architecture is trained to predict brain age and the predicted change is calculated by subtracting two predicted age values for a given pair of images. There is significant variation in individual slopes in both the LILAC and SIRB fitting results, with $p = 6.303e - 6$ and $p = 0.000539$, respectively.

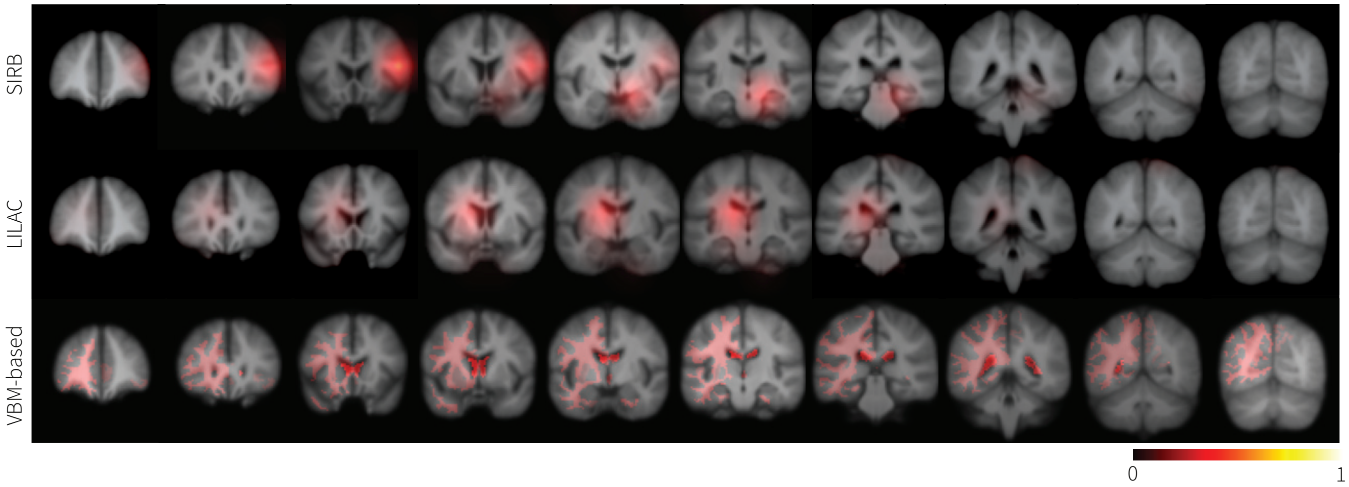

Fig. S6. Population-average modified Grad-CAM peaks were computed over the test set using the maximum time difference pair for each subject. The heatmaps for LILAC and SIRB are overlaid on the averaged image of the entire test set for each dataset. The VBM-based heatmap visualizes the distribution of ROIs with maximal volume change rate.

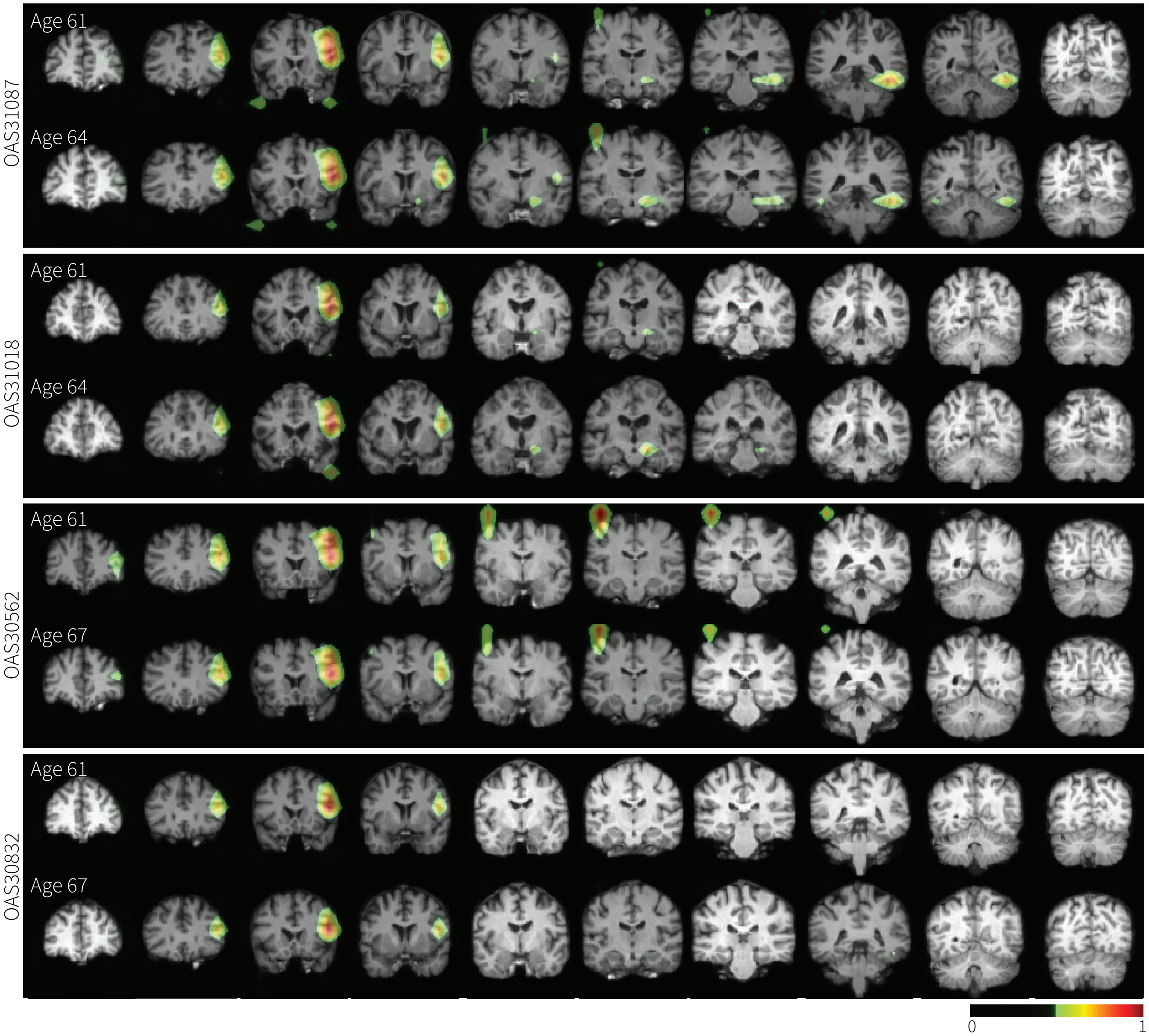

Fig. S7. Localization results of the SIRB trained for predicting age. Although the saliency maps in each panel are from different subjects, they share similar patterns, predominantly highlighting the frontal cortex.

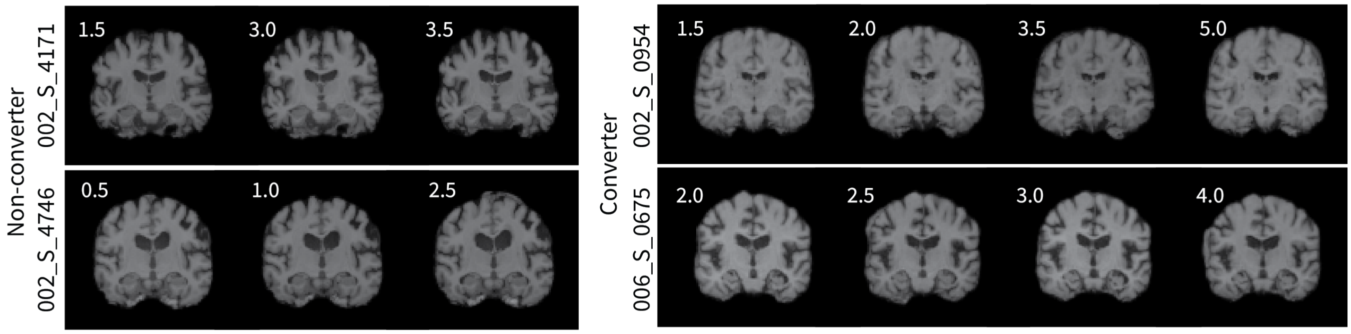

Fig. S8. Representative examples from the mild cognitive impairment (MCI) brain dataset. CDRSB scores are indicated in the top right corner of each image.

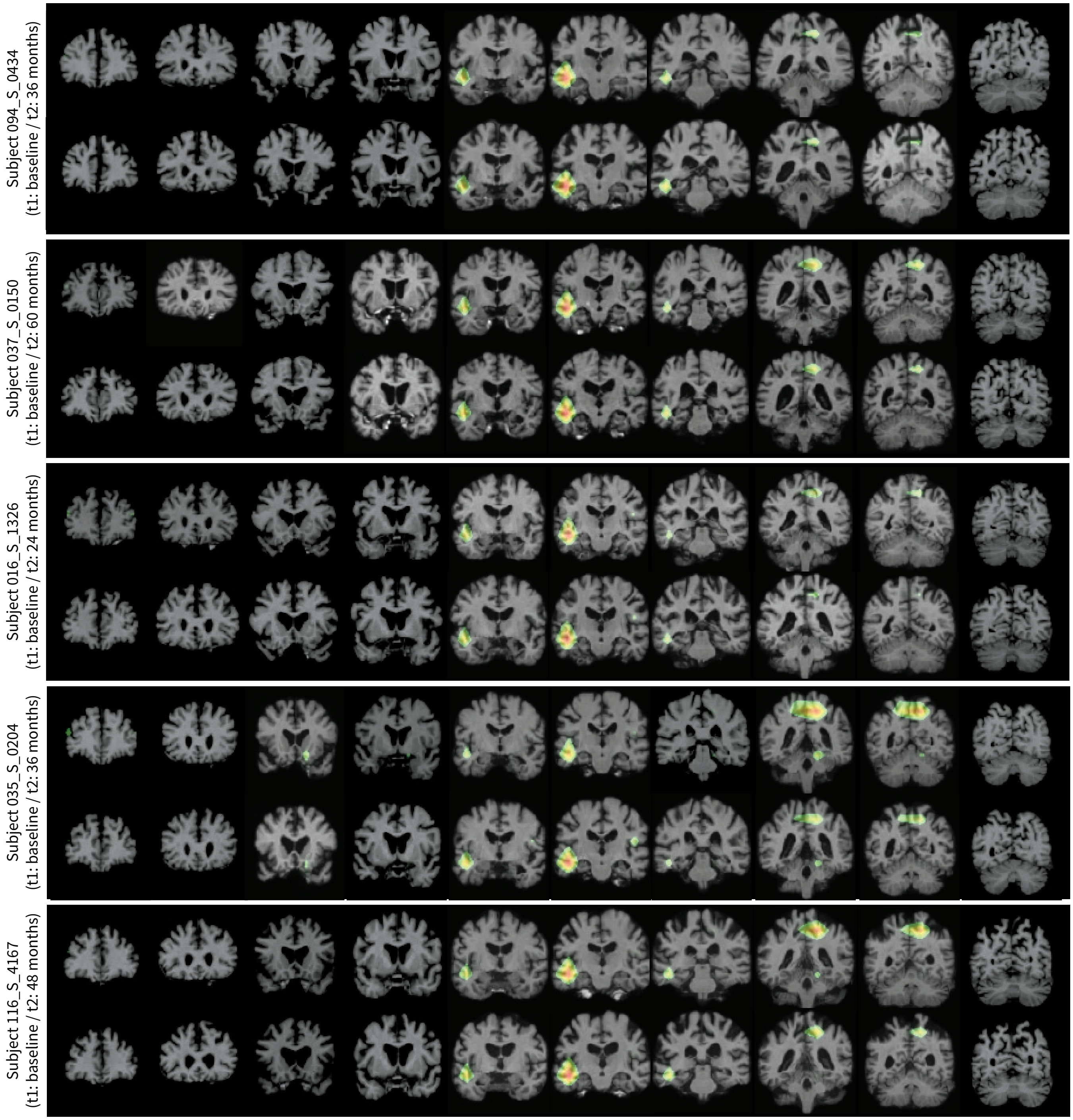

Fig. S9. Localization results of the SIRB trained for predicting CDRSB with age and sex as additional variables. Each panel illustrates the saliency maps from subjects at different time points. The saliency maps share similar patterns, mainly focusing on the temporal lobe.

Table S1. Performance metrics on the MCI test set. Low loss and high R^2 demonstrate the LILAC's explainability with the dataset. Additionally, a high Pearson Correlation Coefficient (PCC) shows the relationship between ground-truth change and predicted value.

	AUC	RMSE	R^2	PCC
Temporal Order	0.956	$-$	0.677	0.753 ($p < 1e-16$)
Time Interval	$-$	1.145	0.706	0.861 ($p < 1e-16$)
CDRSB Change	$-$	1.396	0.684	0.827 ($p < 1e-16$)
CDRSB Change+Time Interval	$-$	1.397	0.683	0.828 ($p < 1e-16$)
CDRSB Change+Time Interval*Sex	$-$	1.415	0.675	0.822 ($p < 1e-16$)

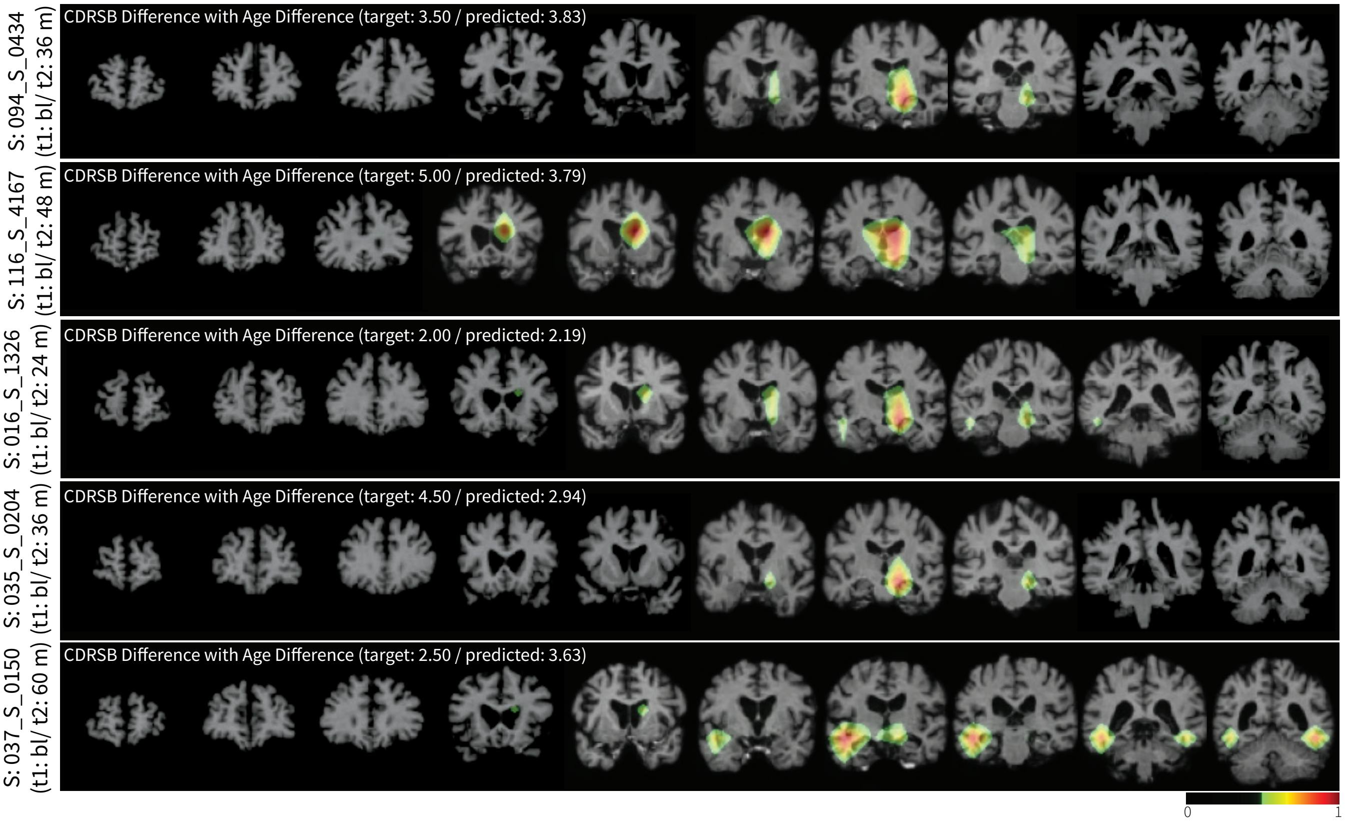

Fig. S10. Additional localization results of the matched subjects of Fig. 6 obtained from LILAC networks trained to predict CDRSB change.

Table S2. Comparison of age prediction models trained on pre-processed vs. raw images.

	# of parameters	augmentation	RMSE	MAE
Preprocessed	105776	[Noise (Gaussian and Gamma), Blur]	1.825	1.087
Preprocessed w/o registration	105776	[Rotation, Translation, Scale, LR flip, Noise (Gaussian and Gamma), Blur]	2.007	1.290
Preprocessed w/o registration	347824	[Rotation, Translation, Scale, LR flip, Noise (Gaussian and Gamma), Blur]	1.887	1.181
Raw	105776	[Rotation, Translation, Scale, LR flip, Noise (Gaussian and Gamma), Blur]	1.863	1.211
Raw	347824	[Rotation, Translation, Scale, LR flip, Noise (Gaussian and Gamma), Blur]	1.682	1.093

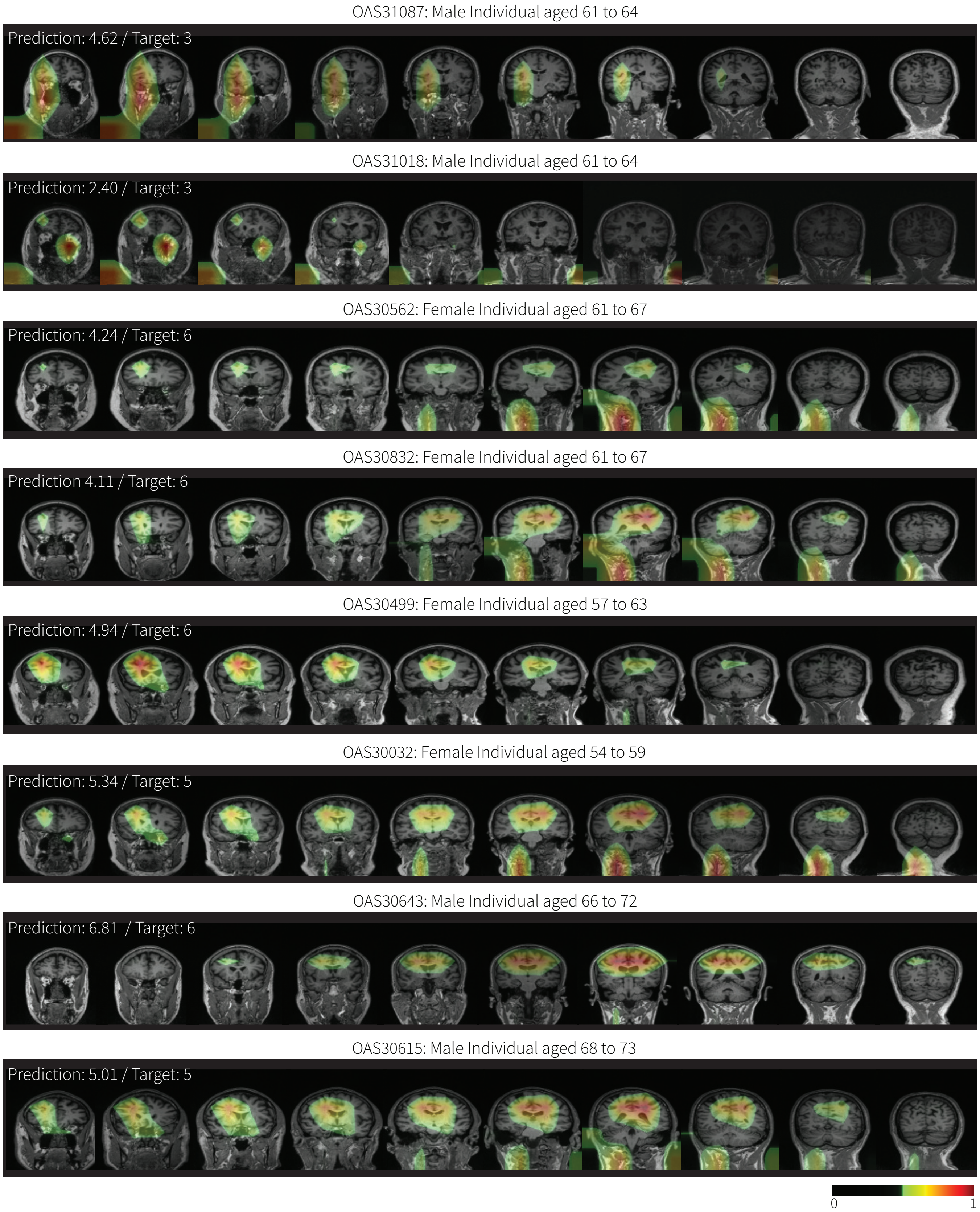

Fig. S11. Modified Grad-CAM results of the network trained without any pre-processing. The images displayed are later time points (t2) of the pairs.

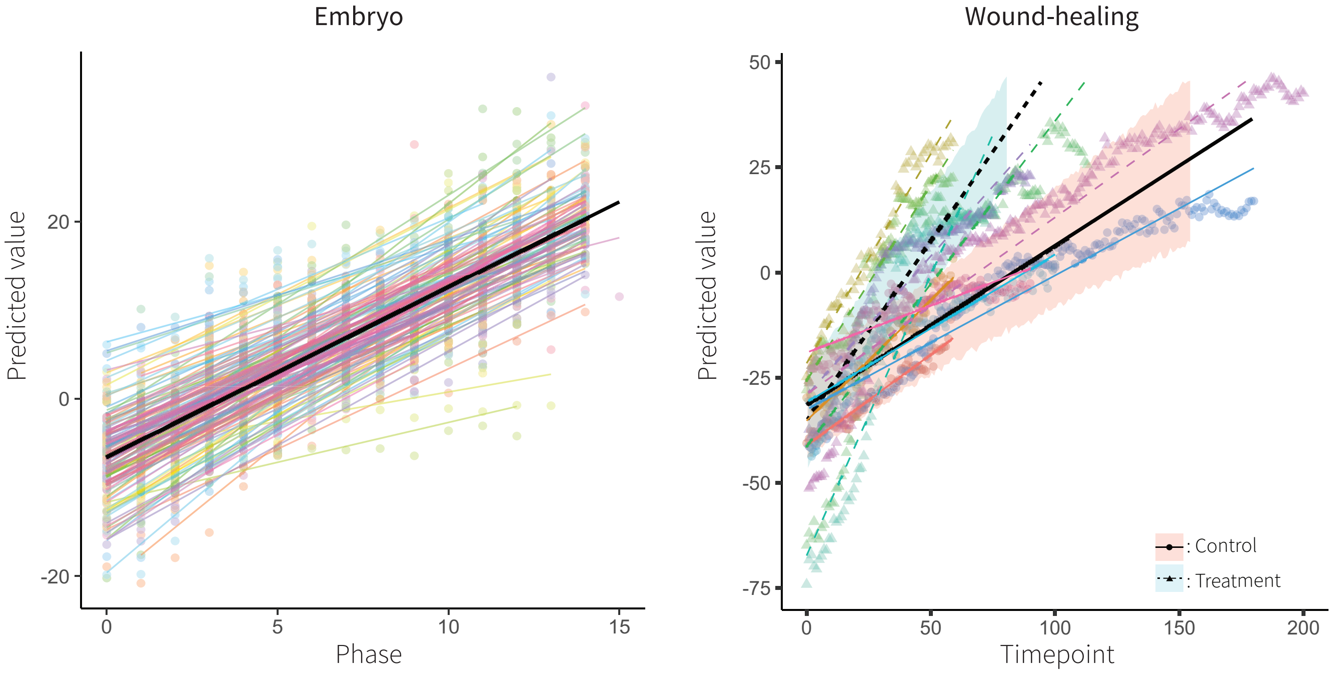

Fig. S12. LME fitting results of the single image predicted values from LILAC networks trained on the temporal ordering tasks. The predicted values successfully capture subject-wise variability and group-wise differences. The group difference in the wound-healing dataset was statistically significant ($p = 0.007154$). There is statistical significance in individual changes in the embryo dataset and the wound-healing dataset (both $p < 2.2e-16$).

Table S3. Comparison of model performance between max pooling and average pooling methods on validation set. Baseline methods involve change prediction using networks trained for single image prediction tasks. We report binary cross-entropy loss for the classification tasks (Embryo and Wound-healing) and mean squared error (MSE) for the regression tasks (Aging Brain and MCI Brain). Embryo and wound-healing models predict temporal ordering, and aging brain models predict temporal intervals. For the MCI brain dataset, the target variable is CDRSB, with age and sex used as additional variables.

	R^2 (↑)		Loss (↓)	
	average pool	max pool	average pool	max pool
Embryo Development	**0.853**	0.842	**0.168**	0.177
Wound-healing	**0.897**	0.885	**0.089**	0.096
Aging Brain	**0.798**	0.733	**1.662**	2.205
MCI Brain	**0.568**	0.559	**1.984**	2.027
Aging Brain Baseline	0.196	**0.570**	6.623	**3.546**
MCI Brain Baseline	0.297	**0.319**	3.230	**3.128**

Table S4. The choices of hyperparameter for training each dataset.

	Batch Size	Input Image Size	Augmentation
Embryo Development	64	224, 224	[Rotation, Translation]
Wound-healing	128	224, 224	[Rotation, Translation, Flip (vertical, horizontal)]
Aging Brain	16	128, 128, 128	[Noise (Gaussian and Gamma), Blur]
MCI Brain	16	128, 128, 128	[Rotation, Translation, LR flip, Noise (Gaussian and Gamma), Blur]

Table S5. Train, Validation, and Test Sample Sizes and Metrics

Dataset	Train Size (Subject/Image/Pair)	Validation Size (Subject/Image/Pair)	Test Size (Subject/Image/Pair)	Metric
Embryo Development	422/5002/61748	141/1640/19990	141/1630/19654	BCE
Wound-healing	14/1515/201153	6/660/85506	11/1087/131395	BCE
Aging Brain	163/472/1552	54/132/346	55/150/442	MSE
MCI Brain	449/2173/11969	150/732/4076	150/711/3819	MSE

Supporting Information Text

Method.

Properties of LILAC. The following properties hold for the proposed LILAC models, where for regression we have $\mathbf{r}(I_i, I_j) = \mathbf{w}^\top((\mathbf{f}(I_1)) - (\mathbf{f}(I_2)))$, with (I_i, I_j) denoting two longitudinal images, and $\mathbf{w}$ representing the weights of the bias-free FC layer. For LILAC-o, this output is fed through a sigmoid σ to produce a probability for the temporal ordering task.

1. Reflexivity: if $I_i = I_j$, then $\mathbf{r}(I_i, I_j) = 0$ and $\sigma(\mathbf{r}(I_i, I_j)) = 0.5$;
2. Antisymmetry: $\mathbf{r}(I_i, I_j) = -\mathbf{r}(I_j, I_i)$ and $\sigma(\mathbf{r}(I_i, I_j)) = 1 - \sigma(\mathbf{r}(I_j, I_i))$; and
3. Additivity: $\mathbf{r}(I_i, I_k) = \mathbf{r}(I_i, I_j) + \mathbf{r}(I_j, I_k)$.
4. Transitivity: if $\mathbf{r}(I_i, I_j) \geq 0$ and $\mathbf{r}(I_j, I_k) \geq 0$, then $\mathbf{r}(I_i, I_k) \geq 0$. Similarly, if $\sigma(\mathbf{r}(I_i, I_j)) \geq 0.5$ and $\sigma(\mathbf{r}(I_j, I_k)) \geq 0.5$, then $\sigma(\mathbf{r}(I_i, I_k)) \geq 0.5$.

We note that these properties are intrinsic to the tasks we consider.

Experimental Setup. Fig. S14 illustrates the architectural details for 2D and 3D input. For all experiments, the models are trained using the Adam optimizer with a learning rate of 0.001. The choices of hyperparameters for each experiment are demonstrated in Table S4. Batches are randomly selected for training in all experiments, except for the MCI brain dataset. For this dataset, the network was initially trained on simpler tasks for the first 10 epochs, given the optimization challenges. During this phase, we began with image pairs separated by more than 50 months and gradually included more pairs every 2 epochs.

Linear Mixed Effect Modeling. The model for predicted change Y can be formulated as follows,

$$y \sim X\beta + Z\alpha + \epsilon, \qquad [1]$$

where $\alpha \sim \mathcal{N}(0, \delta^2 I)$, Z is a design matrix and ϵ is an error term. β is a fixed effects vector and α is a random effects vector. In all experiments, subjects are treated as random effects factors. In temporal difference experiments (i.e., embryo development

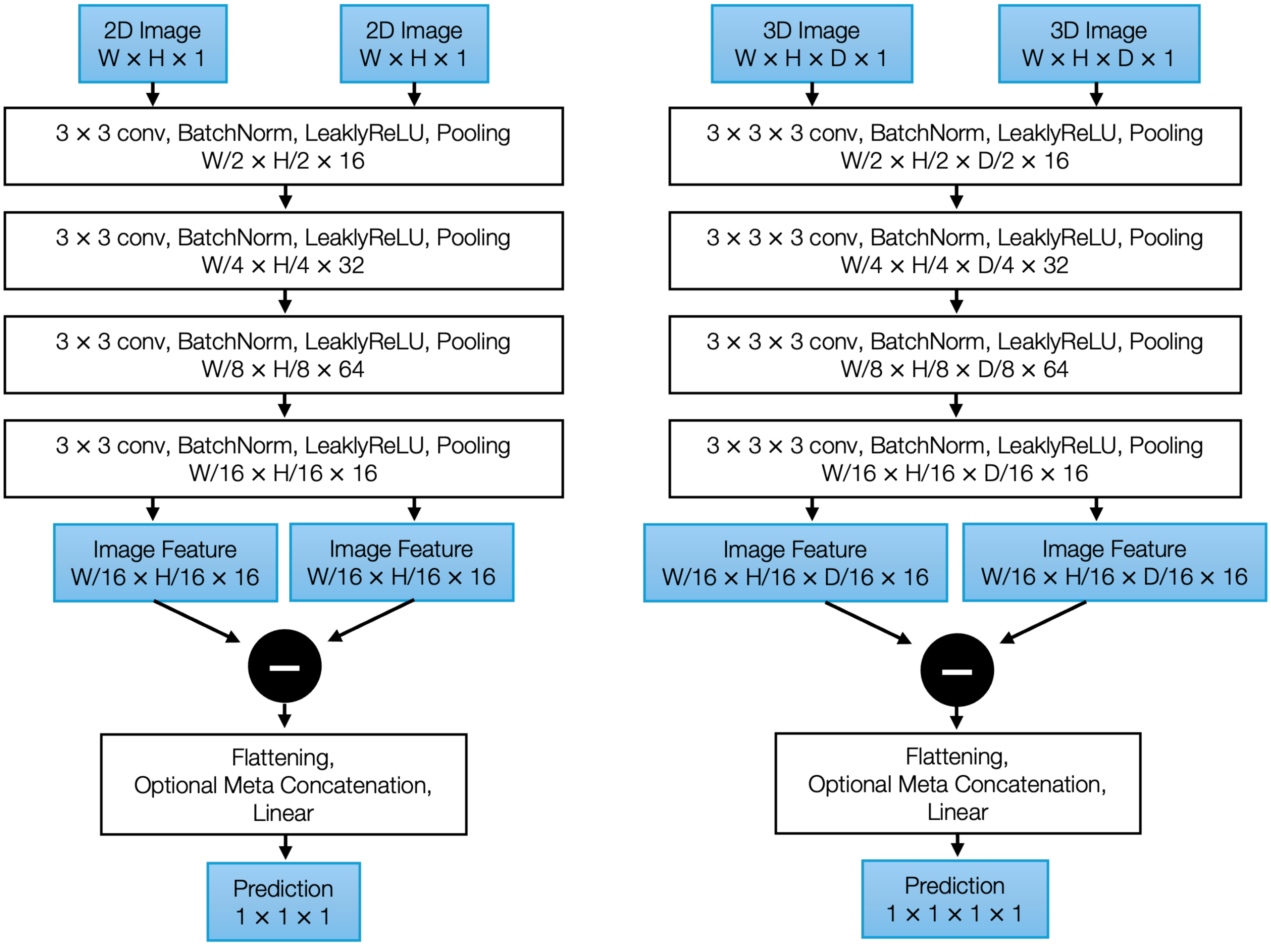

(a) Architecture for 2D input

(b) Architecture for 3D input

Fig. S13. Architectural design for 2D and 3D inputs. W, H, and D represent the width, height, and depth of the input images, respectively.

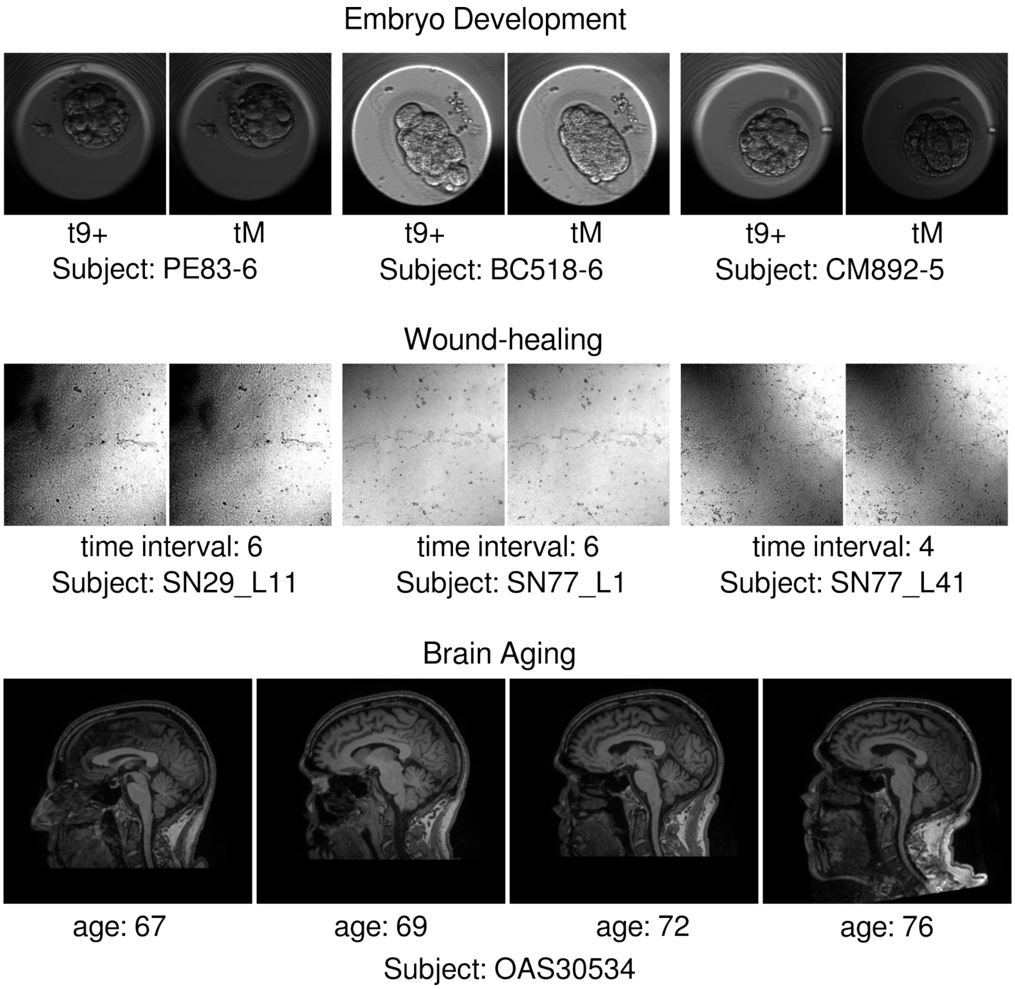

Fig. S14. Example of failure cases.

and aging brain), X is the ground-truth temporal difference t. For the MCI disease progression, t is the change of clinical score. In the wound-healing assay experiments, we assume that the change speed of treatment and control groups is different. We included the group g (treatment/control) as a predictor variable and $X = [t, g]$. For all LME modeling experiments, we utilized *lme4* package (1) using `RStudio` (2).

References

1. D Bates, M Mächler, B Bolker, S Walker, Fitting linear mixed-effects models using lme4. *J. Stat. Softw.* **67**, 1–48 (2015).
2. RStudio Team, *RStudio: Integrated Development Environment for R* (RStudio, PBC., Boston, MA), (2020).

Group Author Information

The Alzheimer's Disease Neuroimaging Initiative Authors. The following authors were part of the Alzheimer's Disease Neuroimaging Initiative: The Data and Publications Committee, in keeping with the publication policies adopted by the ADNI Steering Committee, here provide lists for standardized acknowledgement. The list consists of two parts: Infrastructure Investigators and Site Investigators. Infrastructure Investigators represent the names responsible for leadership and infrastructure. Site Investigators represent the names of individuals at each recruiting site.

I. ADNI 1, GO, 2, 3, 4

Part A: Leadership and Infrastructure

Principal Investigator Michael Weiner, MD University of California, San Francisco Northern California Institute for Research and Education ATRI PI and Director of Coordinating Center Clinical Core Paul Aisen, MD University of Southern California Ronald Petersen, MD, PhD Mayo Clinic, Rochester (co-PI of of Clinical Core) Executive Committee Michael Weiner, MD University of California, San Francisco Paul Aisen, MD University of Southern California Ronald Petersen, MD, PhD Mayo Clinic, Rochester Clifford R. Jack, Jr., MD Mayo Clinic, Rochester William Jagust, MD University of California, Berkeley Susan Landau, PhD University of California, Berkeley Monica Rivera-Mindt, PhD Fordham University; Mt. Sinai Medical Center Ozioma Okonkwo, PhD University of Wisconsin Leslie M. Shaw, PhD University of Pennsylvania Edward B. Lee, MD, PhD University of Pennsylvania Arthur W. Toga, PhD University of California, Los Angeles Laurel Beckett, PhD University of California, Davis Danielle Harvey, PhD University of California, Davis Robert C. Green, MD, MPH Boston University Andrew J. Saykin, PsyD Indiana University Kwangsik Nho, PhD Indiana University Richard J. Perrin, MD, PhD Washington University St. Louis Duygu Tosun, PhD University of California, San Francisco ADNI 4 Private Partner Scientific Board (PPSB) Convened by Alzheimer's Association Pallavi Sachdev, PhD Eisai (Chair, 2023-2024) Data and Publication Committee (DPC) Robert C. Green, MD, MPH Harvard University (Chair) Erin Drake Harvard University Resource Allocation Review Committee Tom Montine, MD, PhD University of Washington (Chair) Cat Conti, BA Northern California Institute for Research and Education Administrative Core Leaders and Key Personnel Michael W. Weiner, MD University of California, San Francisco Rachel Nosheny, PhD University of California, San Francisco Diana Truran Sacrey Northern California Institute for Research and Education Juliet Fockler University of California, San Francisco Melanie J. Miller, PhD Northern California Institute for Research and Education Catherine (Cat) Conti Northern California Institute for Research and Education Winnie Kwang, MA University of California, San Francisco Chengshi Jin, PhD University of California, San Francisco Adam Diaz, MS Northern California Institute for Research and Education Miriam Ashford, PhD Northern California Institute for Research and Education Derek Flenniken Northern California Institute for Research and Education Adrienne Kormos Northern California Institute for Research and Education Clinical Core Leaders and Key Personnel Ronald Petersen, MD, PhD Mayo Clinic, Rochester (Core PI) Paul Aisen, MD University of Southern California (Core PI) Michael Rafii, MD, PhD University of Southern California Rema Raman, PhD University of Southern California Gustavo Jimenez, MBS University of Southern California Michael Donohue, PhD University of Southern California Jennifer Salazar, MBS University of Southern California Andrea Fidell, MPH University of Southern California Virginia Boatwright, BS University of Southern California Justin Robison, MS University of Southern California Caileigh Zimmerman, MS University of Southern California Yuliana Cabrera, BS University of Southern California Sarah Walter, MSc University of Southern California Taylor Clanton, MPH University of Southern California Elizabeth Shaffer, BS University of Southern California Caitlin Webb, BA University of Southern California Lindsey Hergesheimer, BS University of Southern California Stephanie Smith, BS University of Southern California Sheila Ogwang, MPH University of Southern California Olusegun Adegoke, MSc University of Southern California Payam Mahboubi, MPH University of Southern California Jeremy Pizzola, BA University of Southern California Cecily Jenkins, PhD University of Southern California Biostatistics Core Leaders and Key Personnel Laurel Beckett, PhD University of California, Davis (Core PI) Danielle Harvey, PhD University of California, Davis (Core PI) Michael Donohue, PhD University of Southern California Naomi Saito, MS University of California, Davis Adam Diaz, MS Northern California Institute for Research and Education Kedir Adem Hussen, MS University of Southern California Engagement Core Leaders and Key Personnel Ozioma Okonkwo, PhD University of Wisconsin (Core-PI) Monica Rivera-Mindt, PhD Fordham University; Mt. Sinai (Core-PI) Hannatu Amaza University of Wisconsin Mai Seng Thao University of Wisconsin Shaniya Parkins Mt. Sinai Omobolanle Ayo, MBChB, MPH Mt. Sinai Matt Glittenberg University of Wisconsin Isabella Hoang University of Wisconsin Kaori Kubo Germano, PhD Fordham University Joe Strong, PhD University of Wisconsin Trinity Weisensel University of Wisconsin Fabiola Magana University of Wisconsin Lisa Thomas University of Wisconsin Vanessa Guzman, PhD Mt. Sinai Adeyinka Ajayi, MBBS, MPH Mt. Sinai Joseph Di Benedetto, LMSW Mt. Sinai Sandra Talavera, MSW Fordham University MRI Core Leaders and Key

Personnel Clifford R. Jack, Jr., MD Mayo Clinic, Rochester (Core PI) Joel Felmlee, PhD Mayo Clinic, Rochester Nick C. Fox, MD University College London Paul Thompson, PhD UCLA School of Medicine Charles DeCarli, MD University of California, Davis Arvin Forghanian-Arani, PhD Mayo Clinic, Rochester Bret Borowski, RTR Mayo Clinic, Rochester Calvin Reyes Mayo Clinic, Rochester Caitie Hedberg Mayo Clinic, Rochester Chad Ward Mayo Clinic, Rochester Christopher Schwarz, PhD Mayo Clinic, Rochester Denise Reyes Mayo Clinic, Rochester Jeff Gunter, PhD Mayo Clinic, Rochester John Moore-Weiss, PhD Mayo Clinic, Rochester Kejal Kantarci, MD Mayo Clinic, Rochester Leonard Matoush Mayo Clinic, Rochester Matthew Senjem, MS Mayo Clinic, Rochester Prashanthi Vemuri, PhD Mayo Clinic, Rochester Robert Reid, PhD Mayo Clinic, Rochester Ian Malone, PhD University College London Sophia I. Thomopoulos, BS University of Southern California School of Medicine Talia M. Nir, PhD University of Southern California School of Medicine Neda Jahanshad, PhD University of Southern California School of Medicine Alexander Knaack, MS University of California, Davis Evan Fletcher, PhD University of California, Davis Danielle Harvey, PhD University of California, Davis Duygu Tosun-Turgut, PhD University of California, San Francisco Stephanie Rossi Chen, BA. Northern California Institute for Research and Education Mark Choe, BS Northern California Institute for Research and Education Karen Crawford University of Southern California School of Medicine Paul A. Yushkevich, PhD University of Pennsylvania Sandhitsu Das, PhD University of Pennsylvania PET Core Leaders and Key Personnel William Jagust, MD University of California, Berkeley (Core PI) Susan Landau, PhD University of California, Berkeley (Core PI) Robert A. Koeppe, PhD University of Michigan Gil Rabinovici University of California San Francisco Victor Villemagne University of Pittsburgh Brian LoPresti University of Pittsburgh Neuropathology Core Leaders and Key Personnel Richard J. Perrin, MD, PhD Washington University St. Louis (Core PI) John Morris, MD Washington University St. Louis Erin Franklin, MS Washington University St. Louis Haley Bernhardt, BA, R. EEG T. Washington University St. Louis Nigel J. Cairns, PhD, MRCPath Washington University St. Louis Lisa Taylor-Reinwald, BA, HTL (ASCP) Washington University St. Louis Biomarkers Core Leader and Key Personnel Leslie Shaw, PhD UPenn School of Medicine (Core PI) Edward B. Lee, MD, PhD University of Pennsylvania (Core PI) Virginia M.Y. Lee, PhD, MBA UPenn School of Medicine Magdalena Korecka, PhD UPenn School of Medicine Magdalena Brylska, MS UPenn School of Medicine Yang Wan, MS UPenn School of Medicine J.Q. Trojanowki, MD, PhD* UPenn School of Medicine (*former Core PI, deceased) Informatics Core Leader and Key Personnel Arthur W. Toga, PhD University of Southern California (Core PI) Karen Crawford, MLIS University of Southern California Scott Neu, PhD University of Southern California Genetics Core Leader and Key Personnel Andrew J. Saykin, PsyD Indiana University School of Medicine (Core PI) Kwangsik Nho, PhD Indiana University School of Medicine (Core PI) Tatiana M. Foroud, PhD Indiana University School of Medicine (Dir. NCRAD) Taeho Jo, PhD Indiana University School of Medicine Shannon L. Risacher, PhD Indiana University School of Medicine Hannah Craft, MPH Indiana University School of Medicine Liana G. Apostolova, MD Indiana University School of Medicine Kelly Nudelman, PhD NCRAD/Indiana University School of Medicine Kelley Faber, MS, CCRC NCRAD/Indiana University School of Medicine Zoë Potter, BA, CCRP NCRAD/Indiana University School of Medicine Kaci Lacy, MPH, CCRP NCRAD/Indiana University School of Medicine Rima Kaddurah-Daouk, PhD Duke University/AD Metabolomics Consortium Li Shen, PhD University of Pennsylvania ADNI4 Amyloid Disclosure Team Jason Karlawish, MD University of Pennsylvania Claire Erickson, PhD University of Pennsylvania Joshua Grill PhD University of California, Irvine Emily Largent PhD University of Pennsylvania Kristin Harkins MPH University of Pennsylvania Early Project Development Michael W. Weiner, MD UCSF/NCIRE Leon Thal, MD – Past Investigator Zaven Kachaturian, PhD Khachaturian, Radebaugh & Associates (KRA), Inc Richard Frank, MD, PhD General Electric Peter J. Snyder, PhD University of Connecticut Alzheimer's Association's Ronald and Nancy Reagan's Research Institute NIA Neil Buckholtz, PhD National Institute on Aging John K. Hsiao, MD National Institute on Aging Laurie Ryan, PhD National Institute on Aging Susan Molchan, PhD National Institute on Aging/National Institutes of Health ADNI External Scientific Advisory Board (SAB) Zaven Khachaturian, PhD Prevent Alzheimer's Disease 2020 (Chair) Maria Carrillo, PhD Alzheimer's Association William Potter, MD National Institute of Mental Health Lisa Barnes, PhD Rush University Marie Bernard, MD NIA Hector González University of California, San Diego Carole Ho Denali Therapeutics John K. Hsiao, MD NIH Jonathan Jackson, PhD Massachusetts General Hospital Eliezer Masliah, MD NIA Donna Masterman, MD Biogen Ozioma Okonkwo, PhD University of Wisconsin, Madison Richard Perrin, MD, PhD Washington University St. Louis Laurie Ryan, PhD NIA Nina Silverberg, PhD NIA

Part B: Investigators By Site

Oregon Health and Science University: Lisa Silbert, MD Jeffrey Kaye, MD Sylvia White (Salazar), ND Aimee Pierce, MD Amy Thomas, BSN, RN Tera Clay Daniel Schwartz, BA Gillian Devereux, RN, MPH Janet "Janae" Taylor Jennifer Ryan, ND, MS Mike Nguyen Madison DeCapo, BS Yanan Shang, MD University of Southern California: Lon Schneider, MD Cynthia Munoz, MA Diana Ferman, PA Carlota Conant, BS Katherin Martin Kristin Oleary Sonia Pawluczyk, MD Elizabeth Trejo Karen Dagerman Liberty Teodoro, RN Mauricio Becerra Madiha Fairooz, BS Sonia Garrison, MSsc Julia Boudreau, MS Yair Avila, BA University of California–San Diego: James Brewer, MD, PhD Aaron Jacobson Antonio Gama Chi Kim Emily Little, MPH Jennifer Frascino Nichol Ferng Socorro Trujillo, MPH University of Michigan: Judith Heidebrink, MD Robert Koeppe, PhD Steven MacDonald, MD Dariya Malyarenko, Ph.D. Jaimie Ziolkowski, MA, BS, TLLP James O'Connor, MS, RT (R)(MR) Nicole Robert Suzan Lowe Virginia Rogers Mayo Clinic, Rochester: Ronald Petersen, MD, Ph.D. Barbara Hackenmiller Bradley Boeve, MD Colleen Albers, RN Connie Kreuger David Jones, MD David Knopman, MD Hugo Botha, MB, Ch.B. Jessica Magnuson Jonathan Graff-Radford, MD Kerry Crawley, BSW, CCRP Michael Schumacher, CNMT Sanna McKinzie, MS Steven Smith, MS Tascha Helland, BS Val Lowe, MD Vijay Ramanan, MD, PhD Baylor College of Medicine: Valory Pavlik, PhD Jacob Faircloth, BS Jeffrey Bishop, PA Jessica Nath Maria Chaudhary, MAP Maria Kataki, PhD, MD Melissa Yu, MD, FAAN Nathiel Pacini, MA Randall Barker Regan Brooks, BA Ruchi Aggarwal, MD Columbia University Medical Center:

Lawrence Honig, MD, Ph.D. Yaakov Stern, PhD Akiva Mintz, MD Jonathan Cordona, ARRT Michelle Hernandez Washington University, St. Louis: Justin Long, MD Abbey Arnold, NP Alex Groves Anna Middleton, RN Blake Vogler Cierra McCurry Connie Mayo, RN Cyrus Raji, MD, PhD Fatima S. Amtashar, BS Heather Klemp, MSW Heather Nicole Elmore, RN, MSN, ANP-BC, CCRP James Ruszkiewicz, CNMT Jasmina Kusuran Jasmine Stewart Jennifer Horenkamp, RN, BSN Julia Greeson, MS Kara Wever, MA Katie Vo, MD Kelly Larkin, RN Lesley Rao, MD Lisa Schoolcraft, BFA Lora Gallagher Madeline Paczynski, BS, PA-C Maureen McMillan Michael Holt, MSW Nicole Gagliano, BS, RT Rachel Henson, MS Renee LaBarge Robert Swarm, MD Sarah Munie, BSN, RN Serena Cepeda, BS Stacey Winterton, BSN, RN Stephen Hegedus TaNisha Wilson Tanya Harte, FNP-BC Zach Bonacorsi University of Alabama Birmingham: David Geldmacher, MD Amber Watkins, RN Brandi Barger, BSRT Bryan Smelser, MD Charna Bates, MA Cynthia Stover, PENDING Emily McKinley, Gregory Ikner, MA Haley Hendrix, Harold Matthew Cooper, MSN, CRNP, NPC Jennifer Mahaffey, Lindsey Booth Robbins, MSN, CRNP, PNP-C Loren Brown Ashley, RN, BSN Marissa Natelson-Love, MD Princess Carter, RN Veronika Solomon, Mount Sinai School of Medicine: Hillel Grossman, MD Alexandra Groome, BA Allison Ardolino, MA Anthony Kaplan, ARRT, CNMT Faye Sheppard, BS Genesis Burgos-Rivera, BA Gina Garcia-Camilo, MD Joanne Lim, MA Judith Neugroschl, MD Kimberly Jackson, BS Kirsten Evans, BS Laili Soleimani, MD Mary Sano, Ph.D. Nasrin Ghesani, MD Sarah Binder, BS Xiomara Mendoza Apuango, BS Rush University Medical Center: Ajay Sood, MD, PhD Amelia Troutman, MA Kimberly Blanchard, APRN, DNP, NP-C Arlene Richards, Grace Nelson, BA Kirsten Hendrickson, RN, MSN Erin Yurko, Jamie Plenge, BS Victoria Rufo, MS Raj Shah, MD Wein Center: Ranjan Duara, MD Brendan Lynch, CRT Cesar Chirinos, PsyD Christine Dittrich, CRT Debbie Campbell Diego Mejia, CRT Gilberto Perez, CRT Helena Colvee, BS Joanna Gonzalez, PsyD Josalen Gondrez, MS Joshua Knaack Mara Acevedo Maria Cereijo, APRN Maria Greig-Custo, MD Michelle Villar, BS Morris Wishnia Sheryl Detling Warren Barker, MS Johns Hopkins University: Marilyn Albert, Ph.D. Abhay Moghekar Barbara Rodzon Corey Demsky Gregory Pontone, MD Jim Pekar Leonie Farrington, CNRN Martin Pomper Nicole Johnson Tolulope Alo New York University: Martin Sadowski, MD, PhD Anaztasia Ulysse, BA Arjun Masurkar Brittany Marti David Mossa, R.T Emilie Geesey Emily Petrocca, NP Evan Schulze, PhD Jennifer Wong Joseph Boonsiri Sunnie Kenowsky, DVM Tatianne Martinez, NP Veronica Briglall Duke University Medical Center: P. Murali Doraiswamy, MD, MBBS Adaora Nwosu Alisa Adhikari, BS Cammie Hellegers, MA Jeffrey Petrella Olga James, MD Terence Wong Thomas Hawk University of Pennsylvania: Sanjeev Vaishnavi, MD, PhD Hannah McCoubrey, BA Ilya Nasrallah, MD, PhD Rachel Rovere, BA Jeffrey Maneval, MD Elizabeth Robinson, MA Francisco Rivera, MS Jade Uffelman, BS Martha Combs, BS, MS Patricia O'Donnell Sara Manning, MD University of Kentucky: Richard King, MD Alayne Nieto, BSN, RN Amanda Glueck, PhD Anjana Mandal Audrie Swain Bethanie Gamble, PhD, RN Beverly Meacham, RT(R) (MR) Denece Forenback, RN Dorothy Ross, CCRP Elizabeth Cheatham Ellen Hartman Gary Cornell Jordan Harp, PhD Laura Ashe Laura Goins Linda Watts, RN Morgan Yazell Prabin Mandal Regan Buckler, BSN, RN Sylvia Vincent Triana Rudd University of Pittsburgh: Oscar Lopez, MD Ann Arlene Malia Caitlin Chiado, CRNP Cary Zik James Ruszkiewicz, CNMT Kathleen Savage Linda Fenice MaryAnn Oakley, MA Paige C Tacey, M.Ed. Sarah Berman, MD, PhD Sarah Bowser, CRNP Stephen Hegedus Xanthia Saganis University of Rochester Medical Center: Anton Porsteinsson, MD Abigail Mathewson, RN, BSN Asa Widman, BA Bridget Holvey, BS Emily Clark, DO Esmeralda Morales, MS Iris Young, PA-C James Ruszkiewicz, CNMT Kevin Hopkins, BS, CNMT, LNMT Kimberly Martin, RN, BSN Nancy Kowalski, RN, MS Rebecca Hunt, BS Roberta Calzavara, PhD Russell Kurvach, BS, CCRP Stephen D'Ambrosio, PA-C, MPAS University of California, Irvine: Gaby Thai, MD Beatriz Vides, RN, MSN Brigit Lieb, ARRT/CRT Catherine McAdams-Ortiz, MSN, RN, A/GNP Cyndy Toso Ivan Mares, BS Kathryn Moorlach Luter Liu Maria Corona, PhD Mary Nguyen, BA Melanie Tallakson, DNP, FNP-C Michelle McDonnell, PhD Milagros Rangel, BS Neetha Basheer, MD, MBBS Patricia Place, BA Romina Romero, PhD Steven Tam, MD University of Texas Southwestern Medical School: Trung Nguyen, MD, PhD Abey Thomas, ARRT Alexander (Alex) Frolov, MD Alka Khera, MD Amy Browning, BA (Pending) Brendan Kelley (031), MD Courtney Dawson, RT(R) Dana Mathews, MD, Ph.D. Elaine Most, MS (Pending) Elizeva (Ellie) Phillips, CNMT Lynn Nguyen Maribel Nunez Matalin Miller, MS Matthew R. Jones, MA Natalie Martinez, MSN, RN, FNP-BC Rebecca Logan, PA-C Roderick McColl Sari Pham Tiffani Fox, MBA, MS Tracey Moore, BA Emory University: Allan Levey, MD, PhD Abby Brown, NP Andrea Kippels, NP Ashton Ellison, BSPH, ABA Casie Lyons Chadwick Hales, MD, PhD Cindy Parry, BFA Courtney Williams Elizabeth McCorkle, BS Guy Harris, BA Heather Rose, BSN Inara Jooma, BS Jahmila Al-Amin, MS, BS James Lah, MD, PhD James Webster, BS Jessica Swiniarski, MPH, BS Latasha Chapman, BS Laura Donnelly, MPH Lauren Mariotti Mary Locke, BS Phyllis Vaughn, BSN Rachael Penn, BSN, RN Sallie Carpentier, RN, BSN Samira Yeboah, BMSc, R.T.(R) (MR) Sarah Basadre, BMSc, ARRT(R)(MR) Sarah Malakauskas, MS Stefka Lyron, NP Tara Villinger, NP Terra Burney University of Kansas, Medical Center: Jeffrey Burns, MD, MS Ala Abusalim, PA-C Alexandra Dahlgren, BS Alexandria Montero, RN Anne Arthur, BSN, MS, ANP-BC Heather Dooly, BS Katelynn Kreszyn, APRN Katherine Berner, BS Lindsey Gillen, APRN Maria Scanlan, BA Mercedes Madison, BS Nicole Mathis Phyllis Switzer Ryan Townley, MD Samantha Fikru, APRN, MSN, FNP-C Samantha Sullivan, MSW Ella Wright, BS University of California, Los Angeles: Maryam Beigi, MD Anthony Daley Ashley Ko Brittney Luong Glen Nyborg Jessica Morales Kelly Durbin, PhD Lauren Garcia Leila Parand Lorena Macias Lorena Monserratt, PhD Maya Farchi Pauline Wu, DO Robert Hernandez Thao Rodriguez, NP Mayo Clinic, Jacksonville: Neill Graff-Radford, MD, MBBCH, FRCP A'llana Marolt, BS Anton Thomas, BS Deborah Aloszka Ercilia Moncayo, BS Erin Westerhold, RT Gregory Day, MD Kandise Chrestensen, BS Mary Imhansiemhonehi, BS Sanna McKinzie, MS Sochenda Stephens, CCRP Sylvia Grant, CCRC Indiana University: Jared Brosch, MD Amy Perkins, CCRP Aubree Saunders, BS Debra Silberberg Kovac, BS Heather Polson, CNMT Isabell Mwaura, BS Kassandra Mejia, BS Katherine Britt, BS Kathy King, RN Kayla Nichols, BS Kayley Lawrence, BA Lisa Rankin, BSW Martin Farlow, MD Patricia Wiesenauer, MS Robert Bryant, BS Scott Herring, RN Sheryl Lynch, RN Skylar Wilson Traci Day William Korst Yale University School of Medicine: Christopher van Dyck, MD Adam Mecca, MD, PhD Alyssa

Miller, BS Amanda Brennan, LMSE, MSW Amber Khan, MD Audrey Ruan Carol Gunnoud, AS Chelsea Mendonca, MD Danielle Raynes-Goldfinger, BS Elaheh Salardini, MD Elisa Hidalgo, MS, CNMT, EMT, RT (CT) Emma Cooper, BA Erawadi Singh, DO Erin Murphy, BS Jeanine May, APRN, MSN, MHP, CCRP Jesse Stanhope, BS Jessica Lam, BSE Julia Waszak, BS Kimberly Nelsen, BA Kimberly Sacaza, BS Mayer Joshua Hasbani, MD Meghan Donahue, BA Ming-Kai Chen, MD, PhD Nicole Barcelos, MS, MA Paul Eigenberger, MD Robin Bonomi, MD Ryan O'Dell, MD, PhD Sarah Jefferson, MD Siddharth Khasnavis, MD Stephen Smilowitz, MD Susan DeStefano, APRN, MSN Susan Good, APRN Terry Camarro, RT, RN, MRI, APRT Vanessa Clayton, BS Yanis Cavrel, BA YuQuan "Oliver" Lu McGill University, Montreal-Jewish General Hospital: Howard Chertkow, MD Howard Bergman, MD Chris Hosein, M.Ed Sunnybrook Health Sciences, Ontario: Sandra Black, MD Anish Kapadia, MD Aparna Bhan Benjamin Lam, MD, FRCP(c) Christopher Scott, BSc Gillian Gabriel, MA Jennifer Bray, BA, BSW, MSW Ljubica Zotovic, MD Maria Samira Gutierrez Mario Masellis Marjan Farshadi, MD Maurylette Gui, Psych BSc Meghan Mitchell, BSc Rebecca Taylor Ruby Endre, M.R.T Zhala Taghi-Zada University of British Columbia Clinic for AD & Related Disorders Robin Hsiung, MD Carolyn English Ellen Kim, BA Eugene Yau Haley Tong Laura Barlow, RTR/RTMR Lauren Jennings Michele Assaly Paula Nunes, PhD Tahlee Marian Cognitive Neurology St. Joseph's Ontario: Andrew Kertesz, MD John Rogers, MD Dick Trost, PhD Cleveland Clinic Lou Ruvo Center for Brain Health Dylan Wint, MD Charles Bernick, MD Donna Munic, PhD Northwestern University: Ian Grant, MD Aaliyah Korkoyah, BS Ali Raja Allison Lapins, MD Caila Ryan, MS Jelena Pejic Kailey Basham, BS Leena Lukose, BS Loreece Haddad, MS Lucas Quinlan, BS, MLS (ASCP) Nathaniel Houghtaling Premiere Research Inst (Palm Beach Neurology): Carl Sadowsky MD Walter Martinez MD Teresa Villena MD Georgetown University Medical Center: Brigid Reynolds, NP Angelica Forero, MS Carolyn Ward, MSPH Emma Brennan, BS Esteban Figueroa Giuseppe Esposito, MD Jessica Mallory Kathleen Johnson, RN, NP Kathryn Turner, BSN Katie Seidenberg Kelly McCann, BA Margaret Bassett, NP Melanie Chadwick, NP Raymond Scott Turner, MD, PhD Robin Bean, RT Saurabh Sharma, MD Brigham and Women's Hospital: Gad Marshall, MD Aferdita Haviari, BA Alison Pietras, PA-C, ACP Bradley Wallace, BS Catherine Munro, PhD Gladiliz Rivera-Delpin, MA Hadley Hustead, BS Isabella Levesque Jennifer Ramirez, BA Karen Nolan, BS, RT (MR) Kirsten Glennon, RN, CNRN Mariana Palou, BA Michael Erkkinen, MD Nicole DaSilva Pamela Friedman, Psy. D Regina M. Silver, RN Ricardo Salazar, MD Roxxanne Polleys, AA Scott McGinnis (094), MD Seth Gale, MD Tia Hall, BS Tuan Luu Stanford University: Steven Chao, MD Emmeline Lin, BS Jaila Coleman, BA Kevin Epperson, RT(R)(MR) Minal Vasanawala Banner Sun Health Research Institute Alireza Atri, MD, PhD Amy Rangel Brittani Evans Candy Monarrez Carol Cline, LMSW Carolyn Liebsack, RN, BSN, CCRC Daniel Bandy Danielle Goldfarb, MD Debbie Intorcia Jennifer Olgin Kelly Clark Kelsey King, CCRP Kylee York Marina Reade, RN, FNP-C Michael Callan Michael Glass Michaela Johnson, G-ACNP, BC Michele Gutierrez Molly Goddard Nadira Trncic, MD, PhD Parichita Choudhury, MD Priscilla Reyes Serena Lowery Shaundra Hall Sonia Olgin Stephanie de Santiago, RN, NP Boston University: Michael Alosco, PhD Alyssa Ton, BS Amanda Jimenez, MS, EMT-B, CPT Andrew Ellison, MR Technologist Anh Tran, RN Brandon Anderson, RT(N), CNMT Della Carter, MS Donna Veronelli, RTN, CNMT Steven Lenio, MD Eric Steinberg, RN, MSN, CNP Jesse Mez, MD, MS Jason Weller, MD Jennifer Johns, RN Jesse Mez, MD, MS Jessica Harkins, CNMT Alexa Puleio, MS Ina Hoti, BS Jane Mwicigi, MBChB., MPH Alexa Puleio, MS Michael Alosco, PhD Olivia Schultz, BA Mona Lauture, RN Eric Steinberg Ridiane Denis, RN Ronald Killiany, PhD Sarab Singh, CNMT Steven Lenio, MD Wendy Qiu, MD, PhD Ycar Devis, MPH Howard University: Thomas Obisesan, MD, MPH Andrew Stone, MS Debra Ordor, RN, BSN Ifreke Udodong, CRNP Immaculata Okonkwo, DNP, MSN, APRN, FNP-BC Javed Khan, MD Jillian Turner, BS, MS Kyliah Hughes, BS, RMA Oshoze Kadiri, MPH Case Western Reserve University: Charles Duffy, MD, PhD Ariana Moss Katherine Stapleton, LPN Maria Toth (fmr Gross), RN Marianne Sanders, BSN, RN Martin Ayres Melissa Hamski Parianne Fatica, CCRC Paula Ogrocki, PhD Sarah Ash Stacy Pot University of California, Davis Sacramento : Doris Chen, MD Andres Soto Costin Tanase, PhD David Bissig, MD, PhD Hafsanoor Vanya, BA Heather Russell (126), CNMT Hitesh Patel, CNMT Hongzheng Zhang, CCRP Kelly Wallace, CCRP Kristi Ayers, BS Maria Gallegos, BS Martha Forloines, PhD Meghan Sinn Queennie Majorie S Kahulugan, CCRC Richard Isip, RT (R)(N)(CT) Sandra Calderon, MS, RN, FMP-C Talia Hamm, BA, CCRP Parkwood Hospital: Michael Borrie, MD T-Y Lee, PhD Dr Rob Bartha, PhD University of Wisconsin: Sterling Johnson, PhD Sanjay Asthana, MD Cynthia M. Carlsson, MD Banner Alzheimer's Institute: Allison Perrin, MD Pierre Tariot, MD Adam Fleisher, MD Stephanie Reeder, BA Dent Neurologic Institute Horacio Capote, MD Allison Emborsky Anna Mattle, PharmD, MS Bela Ajtai, MD Benjamin Wagner, PA-C Bennett Myers Daryn Slazyk Delaney Fragale, PA-C Erin Fransen, PA Heather Macnamara Jonathan Falletta, PA-C Joseph Hirtreiter, RN Laszlo Mechtler, MD Megan King Michael Asbach, RPA-C Michelle Rainka, Pharm. D., CCRP Richard Zawislak, NP Scott Wisniewski Stephanie O'Malley, PA-C Tatiana Jimenez-Knight Todd Peehler Traci Aladeen, PharmD Vernice Bates Violet Wenner Wisam Elmalik, MD Ohio State University: Douglas W. Scharre, MD Arun Ramamurthy, MD Soumya Bouchachi, MD Maria Kataki, MD, PhD - Past Investigator Rawan Tarawneh, MD - Past Investigator Brendan Kelley, MD - Past Investigator Albany Medical College: Dzintra Celmins, MD Alicia Leader Chris Figueroa Heather Bauerle, NP Katlynn Patterson Michael Reposa Steven Presto Tuba Ahmed Wendy Stewart Hartford Hosp, Olin Neuropsychiatry Research Center: Godfrey D. Pearlson MD Karen Blank, MD Karen Anderson, RN Dartmouth-Hitchcock Medical Center: Robert B. Santulli, MD Eben S. Schwartz, PhD Wake Forest University Health Sciences: Jeff Williamson, MD, MHS, FACP Alicia Jessup, RN Andrea Williams Crystal Duncan Abigail O'Connell, APRN, FNP-C Karen Gagnon Ezequiel Zamora James Bateman Freda Crawford, CNMT Deb Thompson Eboni Walker Jennifer Rowell Mikell White, MHA Phillip "Hunter" Ledford Sarah Bohlman, MSL Susan Henkle, RN Joseph Bottoms, CNMT Lena Moretz, RT(R) CT (MR) Bevan Hoover, BS Michael Shannon Samantha Rogers, PA-C Wendy Baker William Harrison, MD Rhode Island Hospital: Chuang-Kuo Wu, MD Alexis DeMarco, BS Ava Stipanovich, BS, ScM Daniel Arcuri, CNMT, RT(N)(CT) Jan Clark, RN, BSN, CCRC, CSNT Jennifer Davis, PhD Kerstin Doyon, RN, BSN Marie Amoyaw, BA Mauro Veras Acosta, PENDING, BS Ronald Bailey, RT-R, CNMT

Scott Warren, MD Terry Fogerty Victoria Sanborn, PhD Butler Hospital Meghan Riddle, MD Stephen Salloway, MD, MS Paul Malloy, PhD Stephen Correia, PhD University of California San Francisco Charles Windon, MD Morgan Blackburn Howard J. Rosen, MD Bruce L. Miller, MD University of South Florida, Byrd Institute Amanda Smith, MD Ijeoma Mba, MBA, MPH Jenny Echevarria Juris Janavs University of Chicago Emily Roglaski, PhD Meagan Yong Rebecca Devine Eastern Virginia Medical School Hamid Okhravi, MD Charter Health Research Services Edgardo Rivera, MD Teresa Kalowsky Caroline Smith Christina Rosario Houston Methodist Neurological Institute Joseph Masdeu, MD, PhD Richard Le, PharmD Maushami Gurung Barrow Neurological Institute Marwan Sabbagh, MD Angelica Garcia Micah Ellis Slaughter Nadeen Elayan Skieff Acothley Nathan Kline Institute Nunzio Pomara, MD Raymundo Hernando Vita Pomara Chelsea Reichert Ralph Johnson Veterans Administration Health Care Services Olga Brawman-Mintzer, MD Allison Acree Arthur Williams Campbell Long Rebecca Long Vanderbilt University Medical Center Paul Newhouse, MD Sydni Jenee Hill Amy Boegel University of Texas Health, San Antonio Sudha Seshadri, MD Amy Saklad Floyd Jones Rutgers University William Hu, MD, PhD V. Sotelo Gonzalez & Aswad Health Services Yaneicy Gonazalez Rojas, MD Medical University South Carolina Jacobo Mintzer, MD, MBA Crystal Flynn Longmire, PhD Kenneth Spicer, MD, PhD

The group author's contribution is limited to providing the data. As ADNI requires "non-authorship credit on the author byline" as a condition for using ADNI data, we have included the group author alongside the individual author list.
