# Supplementary material for: Learning-based inference of longitudinal image changes: Applications in embryo development, wound healing, and aging brain
Source: Proc Natl Acad Sci U S A. 2025 Feb 20;122(8):e2411492122. doi: 10.1073/pnas.2411492122 (PMC11873959; doi:10.1073/pnas.2411492122)
Supplement: Supplementary file 2 — Dataset S01 (DOCX) [file pnas.2411492122.sd01.docx]

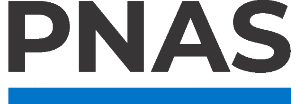


**The Alzheimer’s Disease Neuroimaging Initiative Authors**

The following authors were part of the Alzheimer’s Disease Neuroimaging Initiative:

The Data and Publications Committee, in keeping with the publication policies adopted by the ADNI Steering Committee, here provide lists for standardized acknowledgement. The list consists of two parts: Infrastructure Investigators and Site Investigators. Infrastructure Investigators represent the names responsible for leadership and infrastructure. Site Investigators represent the names of individuals at each recruiting site.

**I. ADNI 1, GO, 2, 3, 4**

**Part A: Leadership and Infrastructure**

Principal Investigator

Michael Weiner, MD University of California, San Francisco

Northern California Institute for Research and Education

ATRI PI and Director of Coordinating Center Clinical Core

Paul Aisen, MD University of Southern California

Ronald Petersen, MD, PhD Mayo Clinic, Rochester (co-PI of of Clinical Core)

Executive Committee

Michael Weiner, MD University of California, San Francisco

Paul Aisen, MD University of Southern California

Ronald Petersen, MD, PhD Mayo Clinic, Rochester

Clifford R. Jack, Jr., MD Mayo Clinic, Rochester

William Jagust, MD University of California, Berkeley

Susan Landau, PhD University of California, Berkeley

Monica Rivera-Mindt, PhD Fordham University; Mt. Sinai Medical Center

Ozioma Okonkwo, PhD University of Wisconsin

Leslie M. Shaw, PhD University of Pennsylvania

Edward B. Lee, MD, PhD University of Pennsylvania

Arthur W. Toga, PhD University of California, Los Angeles

Laurel Beckett, PhD University of California, Davis

Danielle Harvey, PhD University of California, Davis

Robert C. Green, MD, MPH Boston University

Andrew J. Saykin, PsyD Indiana University

Kwangsik Nho, PhD Indiana University

Richard J. Perrin, MD, PhD Washington University St. Louis

Duygu Tosun, PhD University of California, San Francisco

ADNI 4 Private Partner Scientific Board (PPSB) Convened by Alzheimer’s Association

Pallavi Sachdev, PhD Eisai (Chair, 2023-2024)

Data and Publication Committee (DPC)

Robert C. Green, MD, MPH Harvard University (Chair)

Erin Drake Harvard University

Resource Allocation Review Committee

Tom Montine, MD, PhD University of Washington (Chair)

Cat Conti, BA Northern California Institute for Research and Education

Administrative Core Leaders and Key Personnel

Michael W. Weiner, MD University of California, San Francisco

Rachel Nosheny, PhD University of California, San Francisco

Diana Truran Sacrey Northern California Institute for Research and Education

Juliet Fockler University of California, San Francisco

Melanie J. Miller, PhD Northern California Institute for Research and Education

Catherine (Cat) Conti Northern California Institute for Research and Education

Winnie Kwang, MA University of California, San Francisco

Chengshi Jin, PhD University of California, San Francisco

Adam Diaz, MS Northern California Institute for Research and Education

Miriam Ashford, PhD Northern California Institute for Research and Education

Derek Flenniken Northern California Institute for Research and Education

Adrienne Kormos Northern California Institute for Research and Education

Clinical Core Leaders and Key Personnel

Ronald Petersen, MD, PhD Mayo Clinic, Rochester (Core PI)

Paul Aisen, MD University of Southern California (Core PI)

Michael Rafii, MD, PhD University of Southern California

Rema Raman, PhD University of Southern California

Gustavo Jimenez, MBS University of Southern California

Michael Donohue, PhD University of Southern California

Jennifer Salazar, MBS University of Southern California

Andrea Fidell, MPH University of Southern California

Virginia Boatwright, BS University of Southern California

Justin Robison, MS University of Southern California

Caileigh Zimmerman, MS University of Southern California

Yuliana Cabrera, BS University of Southern California

Sarah Walter, MSc University of Southern California

Taylor Clanton, MPH University of Southern California

Elizabeth Shaffer, BS University of Southern California

Caitlin Webb, BA University of Southern California

Lindsey Hergesheimer, BS University of Southern California

Stephanie Smith, BS University of Southern California

Sheila Ogwang, MPH University of Southern California

Olusegun Adegoke, MSc University of Southern California

Payam Mahboubi, MPH University of Southern California

Jeremy Pizzola, BA University of Southern California

Cecily Jenkins, PhD University of Southern California

Biostatistics Core Leaders and Key Personnel

Laurel Beckett, PhD University of California, Davis (Core PI)

Danielle Harvey, PhD University of California, Davis (Core PI)

Michael Donohue, PhD University of Southern California

Naomi Saito, MS University of California, Davis

Adam Diaz, MS Northern California Institute for Research and Education

Kedir Adem Hussen, MS University of Southern California

Engagement Core Leaders and Key Personnel

Ozioma Okonkwo, PhD University of Wisconsin (Core-PI)

Monica Rivera-Mindt, PhD Fordham University; Mt. Sinai (Core-PI)

Hannatu Amaza University of Wisconsin

Mai Seng Thao University of Wisconsin

Shaniya Parkins Mt. Sinai

Omobolanle Ayo, MBChB, MPH Mt. Sinai

Matt Glittenberg University of Wisconsin

Isabella Hoang University of Wisconsin

Kaori Kubo Germano, PhD Fordham University

Joe Strong, PhD University of Wisconsin

Trinity Weisensel University of Wisconsin

Fabiola Magana University of Wisconsin

Lisa Thomas University of Wisconsin

Vanessa Guzman, PhD Mt. Sinai

Adeyinka Ajayi, MBBS, MPH Mt. Sinai

Joseph Di Benedetto, LMSW Mt. Sinai

Sandra Talavera, MSW Fordham University

MRI Core Leaders and Key Personnel

Clifford R. Jack, Jr., MD Mayo Clinic, Rochester (Core PI)

Joel Felmlee, PhD Mayo Clinic, Rochester

Nick C. Fox, MD University College London

Paul Thompson, PhD UCLA School of Medicine

Charles DeCarli, MD University of California, Davis

Arvin Forghanian-Arani, PhD Mayo Clinic, Rochester

Bret Borowski, RTR Mayo Clinic, Rochester

Calvin Reyes Mayo Clinic, Rochester

Caitie Hedberg Mayo Clinic, Rochester

Chad Ward Mayo Clinic, Rochester

Christopher Schwarz, PhD Mayo Clinic, Rochester

Denise Reyes Mayo Clinic, Rochester

Jeff Gunter, PhD Mayo Clinic, Rochester

John Moore-Weiss, PhD Mayo Clinic, Rochester

Kejal Kantarci, MD Mayo Clinic, Rochester

Leonard Matoush Mayo Clinic, Rochester

Matthew Senjem, MS Mayo Clinic, Rochester

Prashanthi Vemuri, PhD Mayo Clinic, Rochester

Robert Reid, PhD Mayo Clinic, Rochester

Ian Malone, PhD University College London

Sophia I. Thomopoulos, BS University of Southern California School of Medicine

Talia M. Nir, PhD University of Southern California School of Medicine

Neda Jahanshad, PhD University of Southern California School of Medicine

Alexander Knaack, MS University of California, Davis

Evan Fletcher, PhD University of California, Davis

Danielle Harvey, PhD University of California, Davis

Duygu Tosun-Turgut, PhD University of California, San Francisco

Stephanie Rossi Chen, BA. Northern California Institute for Research and Education

Mark Choe, BS Northern California Institute for Research and Education

Karen Crawford University of Southern California School of Medicine

Paul A. Yushkevich, PhD University of Pennsylvania

Sandhitsu Das, PhD University of Pennsylvania

PET Core Leaders and Key Personnel

William Jagust, MD University of California, Berkeley (Core PI)

Susan Landau, PhD University of California, Berkeley (Core PI)

Robert A. Koeppe, PhD University of Michigan

Gil Rabinovici University of California San Francisco

Victor Villemagne University of Pittsburgh

Brian LoPresti University of Pittsburgh

Neuropathology Core Leaders and Key Personnel

Richard J. Perrin, MD, PhD Washington University St. Louis (Core PI)

John Morris, MD Washington University St. Louis

Erin Franklin, MS Washington University St. Louis

Haley Bernhardt, BA, R. EEG T. Washington University St. Louis

Nigel J. Cairns, PhD, MRCPath Washington University St. Louis

Lisa Taylor-Reinwald, BA, HTL (ASCP) Washington University St. Louis

Biomarkers Core Leader and Key Personnel

Leslie Shaw, PhD UPenn School of Medicine (Core PI)

Edward B. Lee, MD, PhD University of Pennsylvania (Core PI)

Virginia M.Y. Lee, PhD, MBA UPenn School of Medicine

Magdalena Korecka, PhD UPenn School of Medicine

Magdalena Brylska, MS UPenn School of Medicine

Yang Wan, MS UPenn School of Medicine

J.Q. Trojanowki, MD, PhD* UPenn School of Medicine (*former Core PI, deceased)

Informatics Core Leader and Key Personnel

Arthur W. Toga, PhD University of Southern California (Core PI)

Karen Crawford, MLIS University of Southern California

Scott Neu, PhD University of Southern California

Genetics Core Leader and Key Personnel

Andrew J. Saykin, PsyD Indiana University School of Medicine (Core PI)

Kwangsik Nho, PhD Indiana University School of Medicine (Core PI)

Tatiana M. Foroud, PhD Indiana University School of Medicine (Dir. NCRAD)

Taeho Jo, PhD Indiana University School of Medicine

Shannon L. Risacher, PhD Indiana University School of Medicine

Hannah Craft, MPH Indiana University School of Medicine

Liana G. Apostolova, MD Indiana University School of Medicine

Kelly Nudelman, PhD NCRAD/Indiana University School of Medicine

Kelley Faber, MS, CCRC NCRAD/Indiana University School of Medicine

Zoë Potter, BA, CCRP NCRAD/Indiana University School of Medicine

Kaci Lacy, MPH, CCRP NCRAD/Indiana University School of Medicine

Rima Kaddurah-Daouk, PhD Duke University/AD Metabolomics Consortium

Li Shen, PhD University of Pennsylvania

ADNI4 Amyloid Disclosure Team

Jason Karlawish, MD University of Pennsylvania

Claire Erickson, PhD University of Pennsylvania

Joshua Grill PhD University of California, Irvine

Emily Largent PhD University of Pennsylvania

Kristin Harkins MPH University of Pennsylvania

Early Project Development

Michael W. Weiner, MD UCSF/NCIRE

Leon Thal, MD – Past Investigator

Zaven Kachaturian, PhD Khachaturian, Radebaugh & Associates (KRA), Inc

Richard Frank, MD, PhD General Electric

Peter J. Snyder, PhD University of Connecticut

Alzheimer's Association's Ronald and Nancy Reagan's Research Institute

NIA

Neil Buckholtz, PhD National Institute on Aging

John K. Hsiao, MD National Institute on Aging

Laurie Ryan, PhD National Institute on Aging

Susan Molchan, PhD National Institute on Aging/National Institutes of Health

ADNI External Scientific Advisory Board (SAB)

Zaven Khachaturian, PhD Prevent Alzheimer’s Disease 2020 (Chair)

Maria Carrillo, PhD Alzheimer’s Association

William Potter, MD National Institute of Mental Health

Lisa Barnes, PhD Rush University

Marie Bernard, MD NIA

Hector González University of California, San Diego

Carole Ho Denali Therapeutics

John K. Hsiao, MD NIH

Jonathan Jackson, PhD Massachusetts General Hospital

Eliezer Masliah, MD NIA

Donna Masterman, MD Biogen

Ozioma Okonkwo, PhD University of Wisconsin, Madison

Richard Perrin, MD, PhD Washington University St. Louis

Laurie Ryan, PhD NIA

Nina Silverberg, PhD NIA

**Part B: Investigators By Site**

Oregon Health and Science University:

Lisa Silbert, MD

Jeffrey Kaye, MD

Sylvia White (Salazar), ND

Aimee Pierce, MD

Amy Thomas, BSN, RN

Tera Clay

Daniel Schwartz, BA

Gillian Devereux, RN, MPH

Janet "Janae" Taylor

Jennifer Ryan, ND, MS

Mike Nguyen

Madison DeCapo, BS

Yanan Shang, MD

University of Southern California:

Lon Schneider, MD

Cynthia Munoz, MA

Diana Ferman, PA

Carlota Conant, BS

Katherin Martin

Kristin Oleary

Sonia Pawluczyk, MD

Elizabeth Trejo

Karen Dagerman

Liberty Teodoro, RN

Mauricio Becerra

Madiha Fairooz, BS

Sonia Garrison, MSsc

Julia Boudreau, MS

Yair Avila, BA

University of California--San Diego:

James Brewer, MD, PhD

Aaron Jacobson

Antonio Gama

Chi Kim

Emily Little, MPH

Jennifer Frascino

Nichol Ferng

Socorro Trujillo, MPH

University of Michigan:

Judith Heidebrink, MD

Robert Koeppe, PhD

Steven MacDonald, MD

Dariya Malyarenko, Ph.D.

Jaimie Ziolkowski, MA, BS, TLLP

James O'Connor, MS, RT (R)(MR)

Nicole Robert

Suzan Lowe

Virginia Rogers

Mayo Clinic, Rochester:

Ronald Petersen, MD, Ph.D.

Barbara Hackenmiller

Bradley Boeve, MD

Colleen Albers, RN

Connie Kreuger

David Jones, MD

David Knopman, MD

Hugo Botha, MB, Ch.B.

Jessica Magnuson

Jonathan Graff-Radford, MD

Kerry Crawley, BSW, CCRP

Michael Schumacher, CNMT

Sanna McKinzie, MS

Steven Smith, MS

Tascha Helland, BS

Val Lowe, MD

Vijay Ramanan, MD, PhD

Baylor College of Medicine:

Valory Pavlik, PhD

Jacob Faircloth, BS

Jeffrey Bishop, PA

Jessica Nath

Maria Chaudhary, MAP

Maria Kataki, PhD, MD

Melissa Yu, MD, FAAN

Nathiel Pacini, MA

Randall Barker

Regan Brooks, BA

Ruchi Aggarwal, MD

Columbia University Medical Center:

Lawrence Honig, MD, Ph.D.

Yaakov Stern, PhD

Akiva Mintz, MD

Jonathan Cordona, ARRT

Michelle Hernandez

Washington University, St. Louis:

Justin Long, MD

Abbey Arnold, NP

Alex Groves

Anna Middleton, RN

Blake Vogler

Cierra McCurry

Connie Mayo, RN

Cyrus Raji, MD, PhD

Fatima S. Amtashar, BS

Heather Klemp, MSW

Heather Nicole Elmore, RN, MSN, ANP-BC, CCRP

James Ruszkiewicz, CNMT

Jasmina Kusuran

Jasmine Stewart

Jennifer Horenkamp, RN, BSN

Julia Greeson, MS

Kara Wever, MA

Katie Vo, MD

Kelly Larkin, RN

Lesley Rao, MD

Lisa Schoolcraft, BFA

Lora Gallagher

Madeline Paczynski, BS, PA-C

Maureen McMillan

Michael Holt, MSW

Nicole Gagliano, BS, RT

Rachel Henson, MS

Renee LaBarge

Robert Swarm, MD

Sarah Munie, BSN, RN

Serena Cepeda, BS

Stacey Winterton, BSN, RN

Stephen Hegedus

TaNisha Wilson

Tanya Harte, FNP-BC

Zach Bonacorsi

University of Alabama Birmingham:

David Geldmacher, MD

Amber Watkins, RN

Brandi Barger, BSRT

Bryan Smelser, MD

Charna Bates, MA

Cynthia Stover, PENDING

Emily McKinley,

Gregory Ikner, MA

Haley Hendrix,

Harold Matthew Cooper, MSN, CRNP, NPC

Jennifer Mahaffey,

Lindsey Booth Robbins, MSN, CRNP, PNP-C

Loren Brown Ashley, RN, BSN

Marissa Natelson-Love, MD

Princess Carter, RN

Veronika Solomon,

Mount Sinai School of Medicine:

Hillel Grossman, MD

Alexandra Groome, BA

Allison Ardolino, MA

Anthony Kaplan, ARRT, CNMT

Faye Sheppard, BS

Genesis Burgos-Rivera, BA

Gina Garcia-Camilo, MD

Joanne Lim, MA

Judith Neugroschl, MD

Kimberly Jackson, BS

Kirsten Evans, BS

Laili Soleimani, MD

Mary Sano, Ph.D.

Nasrin Ghesani, MD

Sarah Binder, BS

Xiomara Mendoza Apuango, BS

Rush University Medical Center:

Ajay Sood, MD, PhD

Amelia Troutman, MA

Kimberly Blanchard, APRN, DNP, NP-C

Arlene Richards,

Grace Nelson, BA

Kirsten Hendrickson, RN, MSN

Erin Yurko,

Jamie Plenge, BS

Victoria Rufo, MS

Raj Shah, MD

Wein Center:

Ranjan Duara, MD

Brendan Lynch, CRT

Cesar Chirinos, PsyD

Christine Dittrich, CRT

Debbie Campbell

Diego Mejia, CRT

Gilberto Perez, CRT

Helena Colvee, BS

Joanna Gonzalez, PsyD

Josalen Gondrez, MS

Joshua Knaack

Mara Acevedo

Maria Cereijo, APRN

Maria Greig-Custo, MD

Michelle Villar, BS

Morris Wishnia

Sheryl Detling

Warren Barker, MS

Johns Hopkins University:

Marilyn Albert, Ph.D.

Abhay Moghekar

Barbara Rodzon

Corey Demsky

Gregory Pontone, MD

Jim Pekar

Leonie Farrington, CNRN

Martin Pomper

Nicole Johnson

Tolulope Alo

New York University:

Martin Sadowski, MD, PhD

Anaztasia Ulysse, BA

Arjun Masurkar

Brittany Marti

David Mossa, R.T

Emilie Geesey

Emily Petrocca, NP

Evan Schulze, PhD

Jennifer Wong

Joseph Boonsiri

Sunnie Kenowsky, DVM

Tatianne Martinez, NP

Veronica Briglall

Duke University Medical Center:

P. Murali Doraiswamy, MD, MBBS

Adaora Nwosu

Alisa Adhikari, BS

Cammie Hellegers, MA

Jeffrey Petrella

Olga James, MD

Terence Wong

Thomas Hawk

University of Pennsylvania:

Sanjeev Vaishnavi, MD, PhD

Hannah McCoubrey, BA

Ilya Nasrallah, MD, PhD

Rachel Rovere, BA

Jeffrey Maneval, MD

Elizabeth Robinson, MA

Francisco Rivera, MS

Jade Uffelman, BS

Martha Combs, BS, MS

Patricia O'Donnell

Sara Manning, MD

University of Kentucky:

Richard King, MD

Alayne Nieto, BSN, RN

Amanda Glueck, PhD

Anjana Mandal

Audrie Swain

Bethanie Gamble, PhD, RN

Beverly Meacham, RT(R) (MR)

Denece Forenback, RN

Dorothy Ross, CCRP

Elizabeth Cheatham

Ellen Hartman

Gary Cornell

Jordan Harp, PhD

Laura Ashe

Laura Goins

Linda Watts, RN

Morgan Yazell

Prabin Mandal

Regan Buckler, BSN, RN

Sylvia Vincent

Triana Rudd

University of Pittsburgh:

Oscar Lopez, MD

Ann Arlene Malia

Caitlin Chiado, CRNP

Cary Zik

James Ruszkiewicz, CNMT

Kathleen Savage

Linda Fenice

MaryAnn Oakley, MA

Paige C Tacey, M.Ed.

Sarah Berman, MD, PhD

Sarah Bowser, CRNP

Stephen Hegedus

Xanthia Saganis

University of Rochester Medical Center:

Anton Porsteinsson, MD

Abigail Mathewson, RN, BSN

Asa Widman, BA

Bridget Holvey, BS

Emily Clark, DO

Esmeralda Morales, MS

Iris Young, PA-C

James Ruszkiewicz, CNMT

Kevin Hopkins, BS, CNMT, LNMT

Kimberly Martin, RN, BSN

Nancy Kowalski, RN, MS

Rebecca Hunt, BS

Roberta Calzavara, PhD

Russell Kurvach, BS, CCRP

Stephen D'Ambrosio, PA-C, MPAS

University of California, Irvine:

Gaby Thai, MD

Beatriz Vides, RN, MSN

Brigit Lieb, ARRT/CRT

Catherine McAdams-Ortiz, MSN, RN, A/GNP

Cyndy Toso

Ivan Mares, BS

Kathryn Moorlach

Luter Liu

Maria Corona, PhD

Mary Nguyen, BA

Melanie Tallakson, DNP, FNP-C

Michelle McDonnell, PhD

Milagros Rangel, BS

Neetha Basheer, MD, MBBS

Patricia Place, BA

Romina Romero, PhD

Steven Tam, MD

University of Texas Southwestern Medical School:

Trung Nguyen, MD, PhD

Abey Thomas, ARRT

Alexander (Alex) Frolov, MD

Alka Khera, MD

Amy Browning, BA (Pending)

Brendan Kelley (031), MD

Courtney Dawson, RT(R)

Dana Mathews, MD, Ph.D.

Elaine Most, MS (Pending)

Elizeva (Ellie) Phillips, CNMT

Lynn Nguyen

Maribel Nunez

Matalin Miller, MS

Matthew R. Jones, MA

Natalie Martinez, MSN, RN, FNP-BC

Rebecca Logan, PA-C

Roderick McColl

Sari Pham

Tiffani Fox, MBA, MS

Tracey Moore, BA

Emory University:

Allan Levey, MD, PhD

Abby Brown, NP

Andrea Kippels, NP

Ashton Ellison, BSPH, ABA

Casie Lyons

Chadwick Hales, MD, PhD

Cindy Parry, BFA

Courtney Williams

Elizabeth McCorkle, BS

Guy Harris, BA

Heather Rose, BSN

Inara Jooma, BS

Jahmila Al-Amin, MS, BS

James Lah, MD, PhD

James Webster, BS

Jessica Swiniarski, MPH, BS

Latasha Chapman, BS

Laura Donnelly, MPH

Lauren Mariotti

Mary Locke, BS

Phyllis Vaughn, BSN

Rachael Penn, BSN, RN

Sallie Carpentier, RN, BSN

Samira Yeboah, BMSc, R.T.(R) (MR)

Sarah Basadre, BMSc, ARRT(R)(MR)

Sarah Malakauskas, MS

Stefka Lyron, NP

Tara Villinger, NP

Terra Burney

University of Kansas, Medical Center:

Jeffrey Burns, MD, MS

Ala Abusalim, PA-C

Alexandra Dahlgren, BS

Alexandria Montero, RN

Anne Arthur, BSN, MS, ANP-BC

Heather Dooly, BS

Katelynn Kreszyn, APRN

Katherine Berner, BS

Lindsey Gillen, APRN

Maria Scanlan, BA

Mercedes Madison, BS

Nicole Mathis

Phyllis Switzer

Ryan Townley, MD

Samantha Fikru, APRN, MSN, FNP-C

Samantha Sullivan, MSW

Ella Wright, BS

University of California, Los Angeles:

Maryam Beigi, MD

Anthony Daley

Ashley Ko

Brittney Luong

Glen Nyborg

Jessica Morales

Kelly Durbin, PhD

Lauren Garcia

Leila Parand

Lorena Macias

Lorena Monserratt, PhD

Maya Farchi

Pauline Wu, DO

Robert Hernandez

Thao Rodriguez, NP

Mayo Clinic, Jacksonville:

Neill Graff-Radford, MD, MBBCH, FRCP

A'llana Marolt, BS

Anton Thomas, BS

Deborah Aloszka

Ercilia Moncayo, BS

Erin Westerhold, RT

Gregory Day, MD

Kandise Chrestensen, BS

Mary Imhansiemhonehi, BS

Sanna McKinzie, MS

Sochenda Stephens, CCRP

Sylvia Grant, CCRC

Indiana University:

Jared Brosch, MD

Amy Perkins, CCRP

Aubree Saunders, BS

Debra Silberberg Kovac, BS

Heather Polson, CNMT

Isabell Mwaura, BS

Kassandra Mejia, BS

Katherine Britt, BS

Kathy King, RN

Kayla Nichols, BS

Kayley Lawrence, BA

Lisa Rankin, BSW

Martin Farlow, MD

Patricia Wiesenauer, MS

Robert Bryant, BS

Scott Herring, RN

Sheryl Lynch, RN

Skylar Wilson

Traci Day

William Korst

Yale University School of Medicine:

Christopher van Dyck, MD

Adam Mecca, MD, PhD

Alyssa Miller, BS

Amanda Brennan, LMSE, MSW

Amber Khan, MD

Audrey Ruan

Carol Gunnoud, AS

Chelsea Mendonca, MD

Danielle Raynes-Goldfinger, BS

Elaheh Salardini, MD

Elisa Hidalgo, MS, CNMT, EMT, RT (CT)

Emma Cooper, BA

Erawadi Singh, DO

Erin Murphy, BS

Jeanine May, APRN, MSN, MHP, CCRP

Jesse Stanhope, BS

Jessica Lam, BSE

Julia Waszak, BS

Kimberly Nelsen, BA

Kimberly Sacaza, BS

Mayer Joshua Hasbani, MD

Meghan Donahue, BA

Ming-Kai Chen, MD, PhD

Nicole Barcelos, MS, MA

Paul Eigenberger, MD

Robin Bonomi, MD

Ryan O'Dell, MD, PhD

Sarah Jefferson, MD

Siddharth Khasnavis, MD

Stephen Smilowitz, MD

Susan DeStefano, APRN, MSN

Susan Good, APRN

Terry Camarro, RT, RN, MRI, APRT

Vanessa Clayton, BS

Yanis Cavrel, BA

YuQuan "Oliver" Lu

McGill University, Montreal-Jewish General Hospital:

Howard Chertkow, MD

Howard Bergman, MD

Chris Hosein, M.Ed

Sunnybrook Health Sciences, Ontario:

Sandra Black, MD

Anish Kapadia, MD

Aparna Bhan

Benjamin Lam, MD, FRCP(c)

Christopher Scott, BSc

Gillian Gabriel, MA

Jennifer Bray, BA, BSW, MSW

Ljubica Zotovic, MD

Maria Samira Gutierrez

Mario Masellis

Marjan Farshadi, MD

Maurylette Gui, Psych BSc

Meghan Mitchell, BSc

Rebecca Taylor

Ruby Endre, M.R.T

Zhala Taghi-Zada

University of British Columbia Clinic for AD & Related Disorders

Robin Hsiung, MD

Carolyn English

Ellen Kim, BA

Eugene Yau

Haley Tong

Laura Barlow, RTR/RTMR

Lauren Jennings

Michele Assaly

Paula Nunes, PhD

Tahlee Marian

Cognitive Neurology St. Joseph’s Ontario:

Andrew Kertesz, MD

John Rogers, MD

Dick Trost, PhD

Cleveland Clinic Lou Ruvo Center for Brain Health

Dylan Wint, MD

Charles Bernick, MD

Donna Munic, PhD

Northwestern University:

Ian Grant, MD

Aaliyah Korkoyah, BS

Ali Raja

Allison Lapins, MD

Caila Ryan, MS

Jelena Pejic

Kailey Basham, BS

Leena Lukose, BS

Loreece Haddad, MS

Lucas Quinlan, BS, MLS (ASCP)

Nathaniel Houghtaling

Premiere Research Inst (Palm Beach Neurology):

Carl Sadowsky MD

Walter Martinez MD

Teresa Villena MD

Georgetown University Medical Center:

Brigid Reynolds, NP

Angelica Forero, MS

Carolyn Ward, MSPH

Emma Brennan, BS

Esteban Figueroa

Giuseppe Esposito, MD

Jessica Mallory

Kathleen Johnson, RN, NP

Kathryn Turner, BSN

Katie Seidenberg

Kelly McCann, BA

Margaret Bassett, NP

Melanie Chadwick, NP

Raymond Scott Turner, MD, PhD

Robin Bean, RT

Saurabh Sharma, MD

Brigham and Women's Hospital:

Gad Marshall, MD

Aferdita Haviari, BA

Alison Pietras, PA-C, ACP

Bradley Wallace, BS

Catherine Munro, PhD

Gladiliz Rivera-Delpin, MA

Hadley Hustead, BS

Isabella Levesque

Jennifer Ramirez, BA

Karen Nolan, BS, RT (MR)

Kirsten Glennon, RN, CNRN

Mariana Palou, BA

Michael Erkkinen, MD

Nicole DaSilva

Pamela Friedman, Psy. D

Regina M. Silver, RN

Ricardo Salazar, MD

Roxxanne Polleys, AA

Scott McGinnis (094), MD

Seth Gale, MD

Tia Hall, BS

Tuan Luu

Stanford University:

Steven Chao, MD

Emmeline Lin, BS

Jaila Coleman, BA

Kevin Epperson, RT(R)(MR)

Minal Vasanawala

Banner Sun Health Research Institute

Alireza Atri, MD, PhD

Amy Rangel

Brittani Evans

Candy Monarrez

Carol Cline, LMSW

Carolyn Liebsack, RN, BSN, CCRC

Daniel Bandy

Danielle Goldfarb, MD

Debbie Intorcia

Jennifer Olgin

Kelly Clark

Kelsey King, CCRP

Kylee York

Marina Reade, RN, FNP-C

Michael Callan

Michael Glass

Michaela Johnson, G-ACNP, BC

Michele Gutierrez

Molly Goddard

Nadira Trncic, MD, PhD

Parichita Choudhury, MD

Priscilla Reyes

Serena Lowery

Shaundra Hall

Sonia Olgin

Stephanie de Santiago, RN, NP

Boston University:

Michael Alosco, PhD

Alyssa Ton, BS

Amanda Jimenez, MS, EMT-B, CPT

Andrew Ellison, MR Technologist

Anh Tran, RN

Brandon Anderson, RT(N), CNMT

Della Carter, MS

Donna Veronelli, RTN, CNMT

Steven Lenio, MD

Eric Steinberg, RN, MSN, CNP

Jesse Mez, MD, MS

Jason Weller, MD

Jennifer Johns, RN

Jesse Mez, MD, MS

Jessica Harkins, CNMT

Alexa Puleio, MS

Ina Hoti, BS

Jane Mwicigi, MBChB., MPH

Alexa Puleio, MS

Michael Alosco, PhD

Olivia Schultz, BA

Mona Lauture, RN

Eric Steinberg

Ridiane Denis, RN

Ronald Killiany, PhD

Sarab Singh, CNMT

Steven Lenio, MD

Wendy Qiu, MD, PhD

Ycar Devis, MPH

Howard University:

Thomas Obisesan, MD, MPH

Andrew Stone, MS

Debra Ordor, RN, BSN

Ifreke Udodong, CRNP

Immaculata Okonkwo, DNP, MSN, APRN, FNP-BC

Javed Khan, MD

Jillian Turner, BS, MS

Kyliah Hughes, BS, RMA

Oshoze Kadiri, MPH

Case Western Reserve University:

Charles Duffy, MD, PhD

Ariana Moss

Katherine Stapleton, LPN

Maria Toth (fmr Gross), RN

Marianne Sanders, BSN, RN

Martin Ayres

Melissa Hamski

Parianne Fatica, CCRC

Paula Ogrocki, PhD

Sarah Ash

Stacy Pot

University of California, Davis Sacramento :

Doris Chen, MD

Andres Soto

Costin Tanase, PhD

David Bissig, MD, PhD

Hafsanoor Vanya, BA

Heather Russell (126), CNMT

Hitesh Patel, CNMT

Hongzheng Zhang, CCRP

Kelly Wallace, CCRP

Kristi Ayers, BS

Maria Gallegos, BS

Martha Forloines, PhD

Meghan Sinn

Queennie Majorie S Kahulugan, CCRC

Richard Isip, RT (R)(N)(CT)

Sandra Calderon, MS, RN, FMP-C

Talia Hamm, BA, CCRP

Parkwood Hospital:

Michael Borrie, MD

T-Y Lee, PhD

Dr Rob Bartha, PhD

University of Wisconsin:

Sterling Johnson, PhD

Sanjay Asthana, MD

Cynthia M. Carlsson, MD

Banner Alzheimer's Institute:

Allison Perrin, MD

Pierre Tariot, MD

Adam Fleisher, MD

Stephanie Reeder, BA

Dent Neurologic Institute

Horacio Capote, MD

Allison Emborsky

Anna Mattle, PharmD, MS

Bela Ajtai, MD

Benjamin Wagner, PA-C

Bennett Myers

Daryn Slazyk

Delaney Fragale, PA-C

Erin Fransen, PA

Heather Macnamara

Jonathan Falletta, PA-C

Joseph Hirtreiter, RN

Laszlo Mechtler, MD

Megan King

Michael Asbach, RPA-C

Michelle Rainka, Pharm. D., CCRP

Richard Zawislak, NP

Scott Wisniewski

Stephanie O'Malley, PA-C

Tatiana Jimenez-Knight

Todd Peehler

Traci Aladeen, PharmD

Vernice Bates

Violet Wenner

Wisam Elmalik, MD

Ohio State University:

Douglas W. Scharre, MD

Arun Ramamurthy, MD

Soumya Bouchachi, MD

Maria Kataki, MD, PhD - Past Investigator

Rawan Tarawneh, MD - Past Investigator

Brendan Kelley, MD - Past Investigator

Albany Medical College:

Dzintra Celmins, MD

Alicia Leader

Chris Figueroa

Heather Bauerle, NP

Katlynn Patterson

Michael Reposa

Steven Presto

Tuba Ahmed

Wendy Stewart

Hartford Hosp, Olin Neuropsychiatry Research Center:

Godfrey D. Pearlson MD

Karen Blank, MD

Karen Anderson, RN

Dartmouth-Hitchcock Medical Center:

Robert B. Santulli, MD

Eben S. Schwartz, PhD

Wake Forest University Health Sciences:

Jeff Williamson, MD, MHS, FACP

Alicia Jessup, RN

Andrea Williams

Crystal Duncan

Abigail O'Connell, APRN, FNP-C

Karen Gagnon

Ezequiel Zamora

James Bateman

Freda Crawford, CNMT

Deb Thompson

Eboni Walker

Jennifer Rowell

Mikell White, MHA

Phillip "Hunter" Ledford

Sarah Bohlman, MSL

Susan Henkle, RN

Joseph Bottoms, CNMT

Lena Moretz, RT(R) CT (MR)

Bevan Hoover, BS

Michael Shannon

Samantha Rogers, PA-C

Wendy Baker

William Harrison, MD

Rhode Island Hospital:

Chuang-Kuo Wu, MD

Alexis DeMarco, BS

Ava Stipanovich, BS, ScM

Daniel Arcuri, CNMT, RT(N)(CT)

Jan Clark, RN, BSN, CCRC, CSNT

Jennifer Davis, PhD

Kerstin Doyon, RN, BSN

Marie Amoyaw, BA

Mauro Veras Acosta, PENDING, BS

Ronald Bailey, RT-R, CNMT

Scott Warren, MD

Terry Fogerty

Victoria Sanborn, PhD

Butler Hospital

Meghan Riddle, MD

Stephen Salloway, MD, MS

Paul Malloy, PhD

Stephen Correia, PhD

University of California San Francisco

Charles Windon, MD

Morgan Blackburn

Howard J. Rosen, MD

Bruce L. Miller, MD

University of South Florida, Byrd Institute

Amanda Smith, MD

Ijeoma Mba, MBA, MPH

Jenny Echevarria

Juris Janavs

University of Chicago

Emily Roglaski, PhD

Meagan Yong

Rebecca Devine

Eastern Virginia Medical School

Hamid Okhravi, MD

Charter Health Research Services

Edgardo Rivera, MD

Teresa Kalowsky

Caroline Smith

Christina Rosario

Houston Methodist Neurological Institute

Joseph Masdeu, MD, PhD

Richard Le, PharmD

Maushami Gurung

Barrow Neurological Institute

Marwan Sabbagh, MD

Angelica Garcia

Micah Ellis Slaughter

Nadeen Elayan

Skieff Acothley

Nathan Kline Institute

Nunzio Pomara, MD

Raymundo Hernando

Vita Pomara

Chelsea Reichert

Ralph Johnson Veterans Administration Health Care Services

Olga Brawman-Mintzer, MD

Allison Acree

Arthur Williams

Campbell Long

Rebecca Long

Vanderbilt University Medical Center

Paul Newhouse, MD

Sydni Jenee Hill

Amy Boegel

University of Texas Health, San Antonio

Sudha Seshadri, MD

Amy Saklad

Floyd Jones

Rutgers University

William Hu, MD, PhD

V. Sotelo

Gonzalez & Aswad Health Services

Yaneicy Gonazalez Rojas, MD

Medical University South Carolina

Jacobo Mintzer, MD, MBA

Crystal Flynn Longmire, PhD

Kenneth Spicer, MD, PhD

The group author's contribution is limited to providing the data. As ADNI requires "non-authorship credit on the author byline" as a condition for using ADNI data, we have included the group author alongside the individual author list.
